# COL5A1 Promotes the Progression of Gastric Cancer by Acting as a ceRNA of miR-137-3p to Upregulate FSTL1 Expression

**DOI:** 10.3390/cancers14133244

**Published:** 2022-07-01

**Authors:** Ming Yang, Zhixing Lu, Bowen Yu, Jiajia Zhao, Liang Li, Kaiyu Zhu, Min Ma, Fei Long, Runliu Wu, Gui Hu, Lihua Huang, Jing Chou, Ni Gong, Kaiyan Yang, Xiaorong Li, Yi Zhang, Changwei Lin

**Affiliations:** 1 Department of Gastrointestinal Surgery, The Third Xiangya Hospital of Central South University, Changsha 410013, China; 2204140203@csu.edu.cn (M.Y.); luzhixing@csu.edu.cn (Z.L.); yubowen1992115@csu.edu.cn (B.Y.); 198312163@csu.edu.cn (J.Z.); liliang97116@csu.edu.cn (L.L.); mamin90@csu.edu.cn (M.M.); 188302067@csu.edu.cn (F.L.); wurunliu123@csu.edu.cn (R.W.); hugui22@csu.edu.cn (G.H.); 601500@csu.edu.cn (J.C.); xy3gongni@csu.edu.cn (N.G.); csuky@csu.edu.cn (K.Y.); xiaorongli@csu.edu.cn (X.L.); 2 The Five-Year Program in Clinical Medicine, Xiangya School of Medicine, Central South University, Changsha 410013, China; 8303191615@csu.edu.cn; 3 Center for Experimental Medicine, The Third Xiangya Hospital of Central South University, Changsha 410013, China; lhhuang68@csu.edu.cn

**Keywords:** bioinformatics, COL5A1, miR-137-3p, FSTL1, gastric cancer, immune infiltration

## Abstract

**Simple Summary:**

The expression of a variety of microRNAs (miRNAs) and their target genes in gastric cancer is dysregulated and affects the progression of gastric cancer but has not been fully clarified, while bioinformatics is expected to become a method to reveal the relationship and function between them. Thus, through a variety of bioinformatics analyses and experiments, we confirmed that miR-137-3p played a tumor-suppressive role in gastric cancer, and its target gene COL5A1 could reversely sponge miR-137-3p to relieve its targeted inhibition of FSTL1, which may promote the progression of gastric cancer by affecting immune infiltration. These results may provide new ideas for the treatment and future research of gastric cancer.

**Abstract:**

MicroRNAs (miRNAs) and their target genes have been shown to play an important role in gastric cancer but have not been fully clarified. Therefore, our goal was to identify the key miRNA–mRNA regulatory network in gastric cancer by utilizing a variety of bioinformatics analyses and experiments. A total of 242 miRNAs and 1080 genes were screened from The Cancer Genome Atlas (TCGA) and Gene Expression Omnibus (GEO), respectively. Then, survival-related differentially expressed miRNAs and their differentially expressed target genes were screened. Twenty hub genes were identified from their protein–protein interaction network. After weighted gene co-expression network analysis was conducted, we selected miR-137-3p and its target gene, COL5A1, for further research. We found that miR-137-3p was significantly downregulated and that overexpression of miR-137-3p suppressed the proliferation, invasion, and migration of gastric cancer cells. Furthermore, we found that its target gene, COL5A1, could regulate the expression of another hub gene, FSTL1, by sponging miR-137-3p, which was confirmed by dual-luciferase reporter assays. Knockdown of COL5A1 inhibited the proliferation, invasion, and migration of gastric cancer cells, which could be rescued by the miR-137-3p inhibitor or overexpression of FSTL1. Ultimately, bioinformatics analyses showed that the expression of FSTL1 was highly correlated with immune infiltration.

## 1. Introduction

Gastric cancer (GC) is one of the most common malignant tumors in the digestive tract, and its incidence and mortality rank fifth and fourth, respectively, among all cancers [1]. Patients with GC have no specific clinical symptoms in the early stage, and most patients are in advanced stages when they seek medical treatment, which leads to poor therapeutic effects and prognoses [2]. Currently, there are few clinically targeted drugs for GC, and the effect is not satisfactory. Therefore, it is necessary to identify new potential targets for the development of effective strategies to treat GC.

MicroRNAs (miRNAs) are an important type of gene expression regulator. Mature miRNAs are endogenous, single-stranded, non-coding RNAs that can regulate multiple mRNAs through the miRNA binding sites (also known as miRNA response elements, MREs) [3]. When the expression levels of some target mRNAs containing multiple MREs were upregulated, they can sponge and reduce the content of effective miRNAs in cells. In other words, cancers can reduce expression levels of endogenous miRNAs with a tumor-suppressive effect by upregulating the expression levels of target transcripts [4]. In view of this phenomenon, Pandolfi et al. proposed the competing endogenous RNA (ceRNA) hypothesis, that is, all RNA transcripts, including pseudogenes, lncRNAs, circRNAs, and mRNAs, regulate each other through their common MREs [5]. In recent years, the ceRNA hypothesis has been confirmed to play a crucial role in various tumors, including GC [6,7]. Consequently, as a bridge in RNA communication, miRNAs show an excellent potential to become treatment targets [8,9].

However, this potential has not been well exploited because of individual differences, as well as differences in sample sizes or detection platforms and methods for miRNA microarray analyses. Therefore, the way in which to systematically and effectively identify miRNAs that are dysregulated and play important roles in GC has become a new challenge. Bioinformatics can help to elucidate the biological mysteries resulting from a large amount of complex biological data through the comprehensive utilization of biology, computer science, and information technology [10]. Therefore, bioinformatics is expected to become a method by which to solve the above problem.

It is worth noting that the results of bioinformatics analysis only provide us with a conjecture, which must be verified by experiments. Thus, the aim of this study was to analyze and identify GC-related miRNAs, their target genes, and their ceRNA network by integrating bioinformatics resources, and then carry out experimental verification to identify potential therapeutic targets and to provide a new direction for the treatment of GC.

## 2. Materials and Methods

### 2.1. Tumor Data Download and Differential Expression Analysis

We downloaded miRNA stem loop expression data (miRNA sequencing data; a list of downloaded samples can be found in Table S1), including data from 446 GC samples and 45 normal samples, from The Cancer Genome Atlas (TCGA) database on 26 August 2020, and performed differential analysis using the edgeR package (version 3.38.0) in R software (version 3.6.1) to identify differentially expressed miRNAs (DEMs) [11,12]. Additionally, we downloaded the GSE118916 dataset (microarray gene expression dataset) [13], including 15 GC samples and 15 adjacent non-tumor (normal) samples, from the Gene Expression Omnibus (GEO) database and performed differential analysis using the limma package (version 3.50.0) in R software to obtain differentially expressed genes (DEGs) [14]. The cutoffs were |log_2_FC| > 1.0 and FDR (or P_adj_) < 0.05, where FC denotes the fold change and FDR denotes the false discovery rate. For more details, the code of differential expression analysis can be found in Supplementary Informations.

### 2.2. Cox Proportional Hazards Regression Model Based on DEMs

We used the survival package (version 3.2–13) in R software to perform univariate Cox proportional hazards regression analysis of DEMs to evaluate the impact of a single miRNA on the survival of GC patients and screen survival-related differentially expressed miRNAs (SRDEMs) [15]. The screening cutoff was *p* < 0.05. Then, we used SRDEMs in the multivariate Cox proportional hazards regression analysis and the glmnet package (version 4.1-4) for lasso regression analysis to eliminate some relatively unimportant miRNAs in SRDEMs [16]. The remaining SRDEMs were used to construct a model that could predict the prognosis of GC patients, and the performance of this model was evaluated [17]. The risk scores for each patient were calculated using the following formula: miRNA risk score = β_1_ × exp(miRNA1) + β_2_ × exp(miRNA2) + ... + β_n_×exp(miRNAn), where β is the regression coefficient derived from the multivariate Cox proportional hazards regression model, and exp () is the expression level of SRDEMs [18]. We divided the patients into a high-risk group and a low-risk group according to the median risk score and compared the Kaplan–Meier survival curves of both groups. We then calculated the 1-, 3- and 5-year survival rates of both groups and plotted receiver operating characteristic (ROC) curves to assess whether the prediction ability of the model was reliable [19].

### 2.3. Prediction of Target Genes of SRDEMs and Functional Enrichment Analysis of Differentially Expressed Target Genes (DETGs)

We queried the mature miRNAs of each SRDEM through miRBase on August 28 [20] 2020, predicted their target genes through TargetScan and miRDB [21,22], and regarded the target genes of mature miRNAs belonging to the same SRDEM as the target genes of that SRDEM. Then, we used the VennDiagram package (version 1.7.3) in R software to intersect the target genes of SRDEMs predicted by TargetScan and miRDB and then intersected these overlapping target genes with DEGs to obtain DETGs of each SRDEM [23]. Next, we used the clusterProfiler package (version 4.2.2) in R software for KEGG and GO enrichment analysis of DETGs (cutoff of *p* < 0.05) [24].

### 2.4. Construction and Analysis of PPI Networks with DETGs

The STRING database can help elucidate protein–protein interactions (PPIs) by integrating a large number of known and predicted data between proteins [25]. To study the interaction between DETGs and obtain potential key genes, we constructed their PPI network through the STRING database on August 29, 2020. Genes with significant interactions were screened out on the basis of a confidence score ≥ 0.4, and the filtered results were imported into Cytoscape software (version 3.8.0) for network visualization [26]. We used the cytoHubba plug-in to calculate the maximal clique centrality (MCC) value of each node in the PPI network and selected the genes with the top 20 MCC values as the hub genes [27].

### 2.5. Weighted Gene Co-Expression Network Analysis (WGCNA)

WGCNA package (version 1.71) was used to analyze the coding genes of all samples in GSE118916 [28]. After setting the soft threshold to 12, the co-expression network met the scale-free distribution. We used a dynamic tree-cutting algorithm (module size = 30) to merge genes with similar expression patterns into the same module. Then, we selected three modules containing the most DEGs and DETGs for further analysis. In these three modules, we identified edges with the 250 highest weights and input them into Cytoscape for network visualization.

### 2.6. Cell Culture

The human gastric epithelial cell line GES-1 and GC cell lines AGS, HGC27, MGC803, and MKN45 were purchased from KeyGEN BioTECH (Nanjing, China). GES-1, AGS, and HGC27 cells were cultured in DMEM (Gibco, Waltham, MA, USA), while MGC803 and MKN45 cells were cultured in RPMI-1640 (Gibco). All media were supplemented with 10% fetal bovine serum (FBS, Biological Industries, Beit-Haemek, Israel), 100 KU/L penicillin, and 100 mg/L streptomycin. Cells were maintained in an incubator at 37 °C with 5% CO_2_.

### 2.7. Plasmid Construction

We constructed three types of COL5A1 knockdown plasmids on the basis of the pLKO.1-TRC plasmid and packaged them as lentiviruses. In addition, on the basis of the pCDH plasmid, we constructed plasmids overexpressing the COL5A1 3′UTR, the FSTL1 3′UTR, and FSTL1. COL5A1-wt1/wt2/wt3, COL5A1-mut1/mut2/mut3, and FSTL1 wt/mut firefly luciferase plasmids and Renilla luciferase plasmids were purchased from Shanghai Genechem Co., Ltd. (Shanghai, China).

### 2.8. Transfection

Cells were seeded into 6-well plates and cultured for 24 h. When the density reached 50–60%, Lipofectamine 3000 reagent (Invitrogen, Waltham, MA, USA) was used to transfect the plasmids and miRNA mimics or inhibitor (Biomics, Nantong, China) according to the instructions.

### 2.9. RNA Extraction and Quantitative Real-Time PCR (qRT-PCR)

Total RNA was extracted using an RNA extraction kit (Promega, Bejing, China). cDNAs of COL5A1 and FSTL1 were synthesized using a reverse transcription kit (Yesen, Shanghai, China) and detected using a qRT-PCR kit (Yesen). Reverse transcription and qRT-PCR detection of miR-137-3p were performed with an All-in-One miRNA qRT-PCR Detection Kit (GeneCopoeia, Rockville, MD, USA). All qRT-PCRs were performed on a Roche Lightcycler 480 instrument. With GAPDH or U6 as internal parameters, the relative COL5A1, FSTL1, and miR-137-3p levels were calculated by the 2^−ΔΔCt^ formula. All primers are shown in Table S2.

### 2.10. Western Blotting

Cell proteins were obtained by RIPA buffer and quantified using the BCA method. Then, the proteins were separated by sodium dodecyl sulfate–polyacrylamide gel electrophoresis (SDS-PAGE) and transferred to polyvinylidene fluoride membranes (Millipore, Burlington, MA, USA). After blocking the membrane with 5% skim milk for 1 h at room temperature, the membrane was immunoblotted with primary antibodies overnight and secondary antibodies for 1 h and visualized on an Odyssey CLx Infrared Imaging System (LI-COR Biosciences, Lincoln, NE, USA). COL5A1, FSTL1, and GAPDH antibodies were purchased from Bioworld (Nanjing, China).

### 2.11. Cell Counting Kit-8 (CCK-8) Assay

Cells were seeded at 5000 cells per well and cultured in 96-well plates for 24 h. Then, 10 μL of CCK-8 reagent (Yesen) per well was added, and the mixture was incubated at 37 °C for 2 h in the next 4 days. The relative proliferation was calculated on the basis of the absorbance of each well.

### 2.12. Ethynyldeoxyuridine (EdU) Proliferation Assay

Cells were seeded at 2000 cells per well and cultured in 96-well plates for 24 h. EdU reagent was added, and the mixture was incubated for 2 h according to the instructions provided with the kFluor488Click-iT kit (KeyGen BioTECH). EdU-positive cells were observed and photographed under a fluorescence microscope.

### 2.13. Transwell Migration/Invasion Assay

Transwell chambers (Corning, Glendale, AZ, USA) coated with Matrigel (BD Biosciences, San Jose, CA, USA) were used for the invasion assay, and chambers without Matrigel were used for the migration assay. After seeding 2 × 10^5^ cells per chamber, the upper wells were supplemented with DMEM without serum, and the lower wells were supplemented with DMEM containing 10% FBS. The cells were incubated for 48 h at 37 °C with 5% CO_2_. Then, the chambers were fixed with 4% paraformaldehyde for 30 min and stained with crystal violet dye for 20 min. Finally, the cells were observed and photographed under an inverted microscope after removing nonmigrating or noninvading cells.

### 2.14. Wound Healing Assay

Cells were seeded at 3 × 10^5^ cells per well in 6-well plates and cultured for 24 h. When the cells had completely covered each well, a 10 μL sterile tip was used to scratch the cell surface to create a wound. Photographs were obtained under an inverted microscope at 0 and 48 h to calculate the width of the wound.

### 2.15. Dual-Luciferase Reporter Assay

Cells were seeded at 5 × 10^4^ cells per well in 24-well plates and cultured for 24 h. The dual-luciferase reporter assay was carried out according to the instructions provided with the Dual-Luciferase Reporter Assay Kit (Vazyme, Nanjing, China). Cell lysis and luciferase detection were performed 48 h after transfection. The firefly luciferase activity was normalized to the Renilla luciferase activity. Data are expressed as the percent of luciferase activity in control cells (100%).

### 2.16. ESTIMATE and CIBERSORT

We downloaded the mRNA expression data (mRNA sequencing data; a list of downloaded samples can be found in Table S1) of 375 GC samples and 32 normal samples from the TCGA database on 26 August 2020. The proportion of stromal and immune components in the TME of each sample was estimated with the estimate package (version 1.0.13) in R software and displayed in three forms: stromal score, immune score, and ESTIMATE score, which were positively correlated with the proportion of stromal components, immune components, and their sum, respectively [29]. The higher the scores, the greater the proportion of corresponding components in the TME. In addition, we estimated the proportions of 22 types of immune cells in all GC samples with CIBERSORT method using the IOBR package (version 0.99.9) in R software [30,31]. Only 358 tumor samples with *p* < 0.05 were selected for analysis.

### 2.17. Statistical Analysis

Data are presented as the mean ± standard deviation (SD) from at least three separate experiments. GraphPad Prism 8.0 (San Diego, CA, USA) and R software were used to analyze the data, and a t-test was performed to evaluate differences between the groups. *p* < 0.05 was considered statistically significant: * *p* < 0.05, ** *p* < 0.01, *** *p* < 0.001, **** *p* < 0.0001, # *p* > 0.05.

## 3. Results

### 3.1. Cox Proportional Hazards Regression Model of DEMs

**Figure 1:**
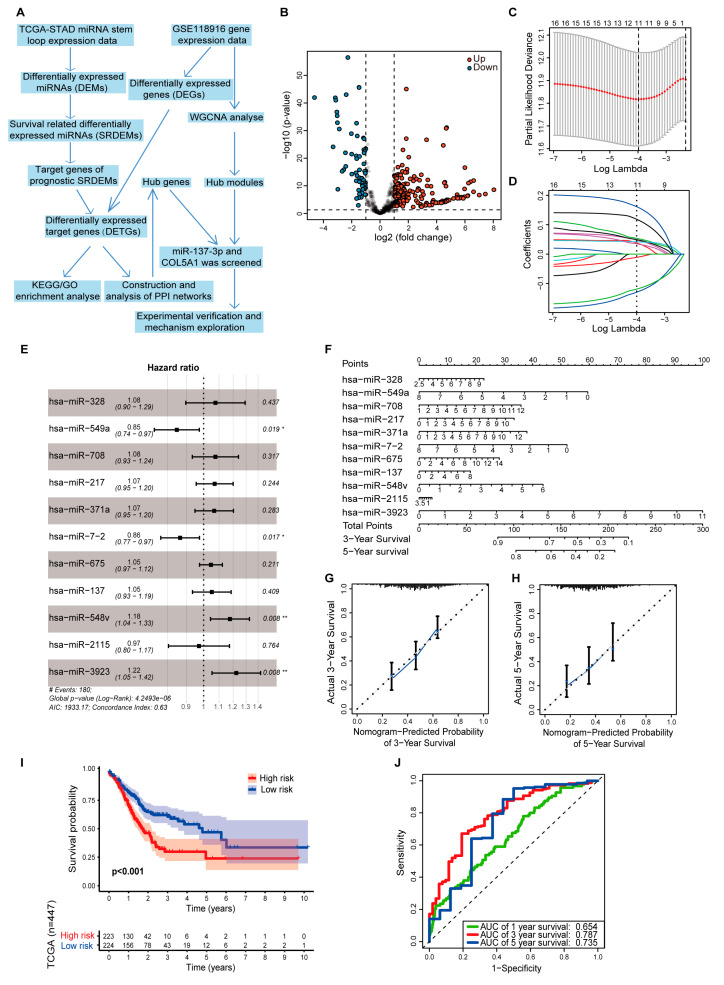
A prognostic model of GC was constructed on the basis of SRDEMs. (**A**) Flow chart of this study. (**B**) Volcano plot of DEMs in TCGA. Red represents upregulation, and blue represents downregulation. (**C**,**D**) Lasso regression analysis was conducted. The vertical dotted line in subfigure C corresponds to the penalty value of the lowest point (the upper coordinate corresponding to the lowest point of the red curve). Different curves in subfigure D represent different miRNAs. Make a vertical line at the position of the this penalty value in subfigure D, then the number of intersection points is the number of variables included in the final model, and the ordinate of the corresponding intersection point is the regression coefficient of the variable. (**E**) Forest plot of multivariate Cox analysis based on SRDEMs. (**F**) Nomogram of the prognostic model. According to the contribution of each factor in the model (the regression coefficient), each value of factor is scored, and then the total score is obtained by adding each score. The predicted outcome is calculated through the functional conversion relationship between each total score and the individual outcome. Calibration charts of 3-year (**G**) or 5-year (**H**) survival based on the prognostic model. (**I**) Kaplan–Meier survival curves for high-risk and low-risk groups. (**J**) The 1-, 3-, and 5-year ROC curves of the model were analyzed.

A flow chart of this study is shown in Figure 1A. By analyzing the TCGA data, we identified 242 DEMs with statistical significance, which consisted of 178 upregulated miRNAs and 64 downregulated miRNAs (Figure 1B). To determine which miRNAs significantly affected the survival of GC patients, we identified 18 SRDEMs by univariate Cox proportional hazards regression analysis (*p* < 0.05, Table 1). Then, we used these SRDEMs for multivariate Cox proportional hazards regression analysis. Lasso regression analysis was utilized to eliminate some relatively unimportant miRNAs in SRDEMs, and 11 SRDEMs (hsa-miR-328, hsa-miR-549a, hsa-miR-708, hsa-miR-217, hsa-miR-371a, hsa-miR-7-2, hsa-miR-675, hsa-miR-137, hsa-miR-548v, hsa-miR-2115, hsa-miR-3923) were screened out to construct a prognostic model (Figure 1C,D). The risk score was calculated as follows: miRNA risk score = (0.07279 × hsa-miR-328) + (−0.16586 × hsa-miR-549a) + (0.07277 × hsa-miR-708) + (0.06800 × hsa-miR-217) + (0.06526 × hsa-miR-371a) + (−0.14550 × hsa-miR-7-2) + (0.04510 × hsa-miR-675) + (0.05038 × hsa-miR-137) + (0.16243 × hsa-miR-548v) + (−0.02891 × hsa-miR-2115) + (0.20189 × hsa-miR-3923). A forest plot of multivariate Cox analysis is shown in Figure 1E, and the nomogram of the model is shown in Figure 1F. The calibration charts for predicting the 3- and 5-year survival rates of GC patients from TCGA based on the nomogram are shown in Figure 1G,H, respectively. The 3- and 5-year survival rates predicted by the nomogram were essentially consistent with the actual 3- and 5-year survival rates of GC patients. Kaplan–Meier survival analysis showed a significant difference in survival between the high- and low-risk groups (*p* < 0.001; Figure 1I). The areas under the 1-, 3-, and 5-year ROC curves were 0.654, 0.787, and 0.735, respectively, indicating that the model could effectively predict the prognosis of GC patients (Figure 1J).

### 3.2. Prediction of Target Genes of SRDEMs and Functional Enrichment Analysis of DETGs

**Figure 2:**
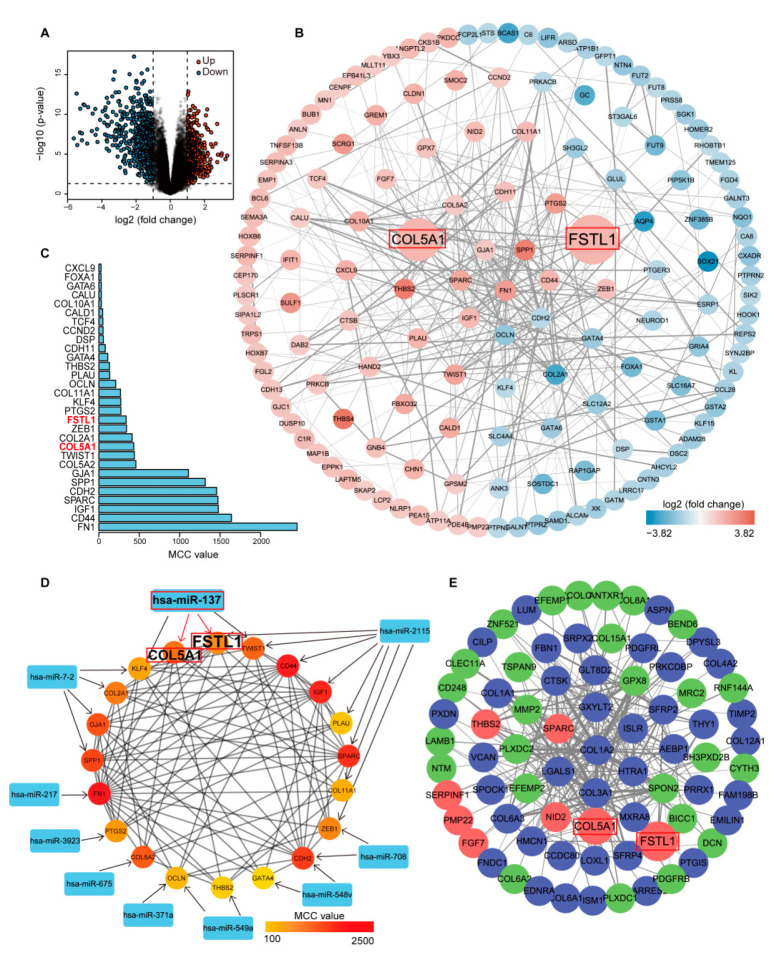
Mining of hub genes in GC. (**A**) Volcano plot of DEGs in GSE118916. (**B**) PPI network of DETGs. Red represents upregulation, and blue represents downregulation. The color of the node deepens as the value of |log_2_FC| increases. (**C**) Genes with the top 30 MCC values in the PPI network. (**D**) The PPI network of 20 hub genes and the SRDEMs that target these genes. The color of the node deepens from yellow to red as the MCC value increases. (**E**) Weighted gene co-expression network of the top 250 edges in the dark gray module. Blue represents DEGs, red represents DETGs, and green represents other genes. In this figure, COL5A1, FSTL1, and miR-137-3p are highlighted.

Since miRNAs generally function by targeting genes, we used two independent online tools (TargetScan and miRDB) to predict the target genes of 11 SRDEMs. As shown in Figure S1, after intersection of the prediction results from two websites, 114, 408, 496, 240, 274, 695, 279, 851, 497, 1303, and 196 target genes of hsa-miR-328, hsa-miR-549a, hsa-miR-708, hsa-miR-217, hsa-miR-371a, hsa-miR-7-2, hsa-miR-675, hsa-miR-137, hsa-miR-548v, hsa-miR-2115, and hsa-miR-3923 were determined, respectively. In addition, analysis of the GSE118916 dataset identified 1080 DEGs with statistical significance, including 474 upregulated genes and 606 downregulated genes (Figure 2A). By intersecting 1080 DEGs with the predicted target genes, 6, 20, 38, 13, 13, 31, 23, 56, 26, 82 and 13 DETGs of these SRDEMs were obtained, yielding a total of 321 target relationships and 233 DETGs (Table 2, Figure S2). To further reveal the potential biological functions of these 233 DETGs, we performed KEGG and GO enrichment analyses. The results of the KEGG analysis showed that these DETGs were mainly enriched in the PI3K-Akt signaling pathway, focal adhesion, proteoglycan in cancer, extracellular matrix, and other cancer-related pathways (Figure S3A). The results of the GO analysis showed that these DETGs were mainly involved in biological processes such as cell proliferation, cell migration, extracellular matrix, and angiogenesis (Figure S3B). These results indicate that these DETGs may play an important role in the development of GC.

### 3.3. Identification of Hub Genes in DETGs

A SRDEM can target multiple DETGs, and a DETG can also be targeted by multiple SRDEMs, indicating that there are correlations between these DETGs. To clarify their interactions, we input all DETGs into the STRING database to construct the PPI network (Figure 2B), which contained 148 nodes and 355 edges (several discrete nodes were eliminated). Each node in the network represents a gene, and the connections between nodes symbolize the interactions between proteins encoded by corresponding genes. The MCC value can be used to evaluate the importance of each node and help identify the hub genes in the network. Therefore, we used the cytoHubba plug-in to calculate the MCC value of each node. The nodes with the top 30 MCC values are shown in Figure 2C. Then, we regarded the genes with the top 20 MCC values as the hub genes in the network: FN1, CD44, IGF1, SPARC, CDH2, SPP1, GJA1, COL5A2, TWIST1, COL5A1, COL2A1, ZEB1, FSTL1, PTGS2, KLF4, COL11A1, OCLN, PLAU, THBS2, and GATA4. The PPI network of these 20 hub genes is shown in Figure 2D.

### 3.4. WGCNA Identification of the Hub Module

WGCNA is a method that uses a large amount of gene expression data to construct the correlations between genes, which can be used to analyze the gene expression patterns of multiple samples and can cluster genes with similar expression patterns. The higher the correlation between genes, the higher their co-expression degree. Therefore, we performed WGCNA to analyze the GSE118916 dataset to determine a class of genes playing an important role in GC. With different power values ranging from 1 to 20, we determined that the best soft threshold was 12 (Figure S4A), and 32 different modules were then generated according to the independence and average connectivity of the network. The co-expression degree of genes in the same module was high, while the co-expression degree of genes from different modules was low (Figure S4B). Among them, the top five modules containing the most DEGs and DETGs are shown in Table S3. Moreover, we constructed weighted gene co-expression networks for the top three modules (bisque4, blue, dark gray) using the edges with the 250 highest weights in each module (Figure 2E and Figure S4C,D). We found that although the bisque4 module contained the most DEGs and DETGs, it contained only four hub genes, while the dark gray module (Figure 2E) had fewer DEGs and DETGs but contained eight hub genes. Thus, we identified this module as the hub module. Interestingly, we found that collagen genes (COL genes) accounted for a very high proportion of the hub genes in the PPI network (COL5A2, COL5A1, COL2A1, COL11A1), while COL genes also frequently appeared in the dark gray module, in which only COL5A1 was located in the center, suggesting an important role of COL5A1. Bioinformatics analyses showed that COL5A1 was highly expressed in GC, while previous prediction analyses showed that the mature miRNA regulating COL5A1 was only miR-137-3p (Table 2, Figure 2D), which was expressed at low levels in GC, suggesting that miR-137-3p and COL5A1 were involved in the progression of GC. Therefore, we selected miR-137-3p and COL5A1 for further experimental verification.

### 3.5. MiR-137-3p Suppressed the Proliferation, Invasion, and Migration of GC Cells

**Figure 3:**
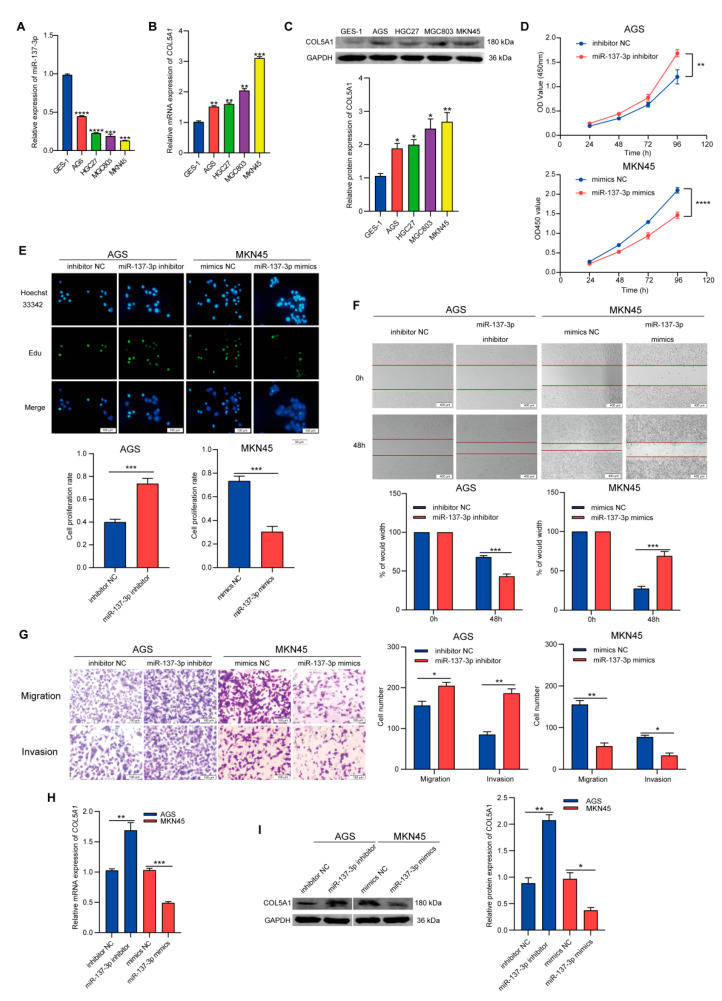
MiR-137-3p played a tumor-suppressive role in GC. (**A**) The expression level of miR-137-3p in GC cells was detected by qRT-PCR. The expression level of COL5A1 in GC cells was detected by qRT-PCR (**B**) and Western blotting (**C**). CCK-8 assays (**D**) and EdU assays (**E**) were carried out to evaluate the cell proliferation ability. Wound healing assays (**F**) and Transwell assays (**G**) were conducted to evaluate the migration and invasion ability in different groups. qRT-PCR (**H**) and Western blotting (**I**) were used to detect the mRNA and protein levels of COL5A1 after transfection of miR-137-3p mimics or inhibitor. Full uncropped figures of Western blotting can be found in Figures S7 and S8. Data are presented as mean ± SD of three independent experiments. * *p* < 0.05; ** *p* < 0.01; *** *p* < 0.001; **** *p* < 0.0001.

First, we verified the expression of miR-137-3p and COL5A1 in GC cells. By comparing the expression levels of miR-137-3p (Figure 3A) and COL5A1 (Figure 3B,C) in GES-1 and four GC cell lines (AGS, HGC27, MGC803, MKN45), we obtained results consistent with the bioinformatics analyses, that is, miR-137-3p was expressed at low levels in GC, while COL5A1 was highly expressed. Then, we transfected miR-137-3p inhibitor into AGS cells with high expression of miR-137-3p and miR-137-3p mimics into MKN45 cells with low expression of miR-137-3p (Figure S5A). Using CCK-8 (Figure 3D) and EdU assays (Figure 3E), we confirmed that the proliferation of AGS cells was enhanced after transfection with the miR-137-3p inhibitor, while the proliferation of MKN45 cells was decreased after transfection with miR-137-3p mimics. We also found that the migration and invasion ability of AGS cells were enhanced after transfection with the miR-137-3p inhibitor, while the opposite results were observed in MKN45 cells transfected with miR-137-3p mimics (Figure 3F,G). These results suggested that miR-137-3p played a tumor-suppressive role in GC.

**Figure 4:**
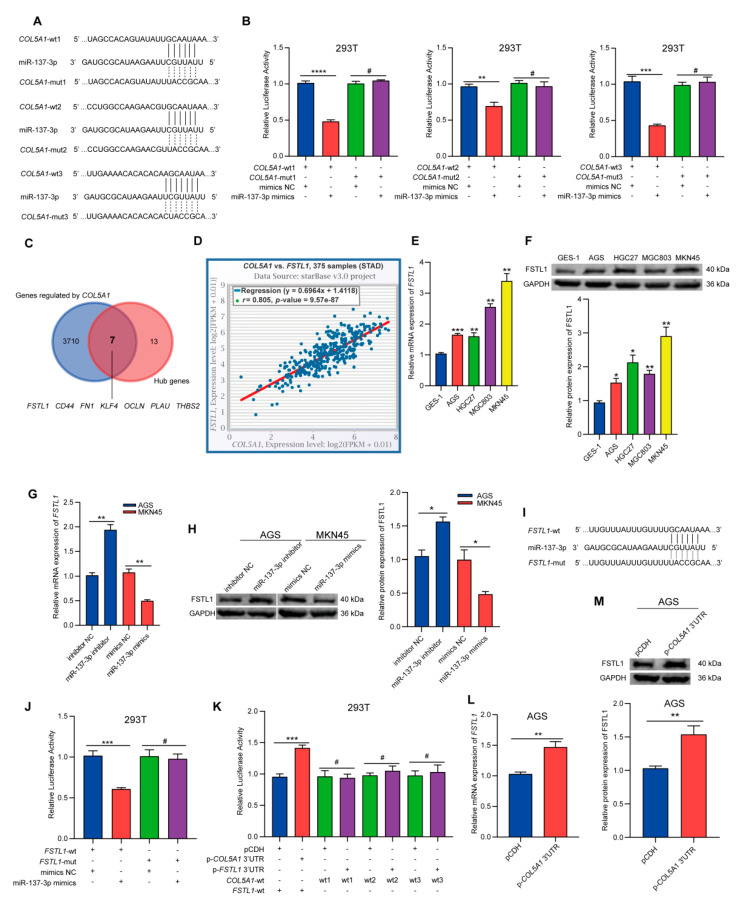
COL5A1 regulated FSTL1 by competitive binding to miR-137-3p through a ceRNA mechanism. (**A**) Construction of the COL5A1-wt/mut luciferase plasmid for 3 binding sites of miR-137-3p. (**B**) Dual-luciferase reporter assays were conducted to verify the binding between miR-137-3p and COL5A1. (**C**) The intersection of genes regulated by COL5A1 (blue) and the hub genes (red). (**D**) Correlation analysis of COL5A1 and FSTL1 in GC from TCGA. The expression level of FSTL1 was assessed by qRT-PCR (**E**) and Western blotting (**F**). qRT-PCR (**G**) and Western blotting (**H**) were performed to detect the mRNA and protein levels of FSTL1 after transfection with miR-137-3p mimics or inhibitor. (**I**) Construction of the FSTL1-wt/mut luciferase plasmid for the binding site of miR-137-3p. Dual-luciferase reporter assays were conducted to verify the binding between miR-137-3p and FSTL1 (**J**) and to confirm that the COL5A1 3′UTR could competitively bind miR-137-3p (**K**). The expression level of FSTL1 was assessed by qRT-PCR (**L**) and Western blotting (**M**) after transfection with the COL5A1 3′UTR in AGS cells. Full uncropped figures of Western blotting can be found in Figures S9– S11. Data are presented as mean ± SD of three independent experiments. # *p* > 0.05; * *p* < 0.05; ** *p* < 0.01; *** *p* < 0.001; **** *p* < 0.0001.

To further clarify whether miR-137-3p could target and regulate the expression of COL5A1, we detected the effect of changing the expression level of miR-137-3p on the expression level of COL5A1. Compared with the control group, the expression of COL5A1 increased after transfection of the miR-137-3p inhibitor, while it decreased significantly after transfection of the miR-137-3p mimics, indicating that COL5A1 was regulated by miR-137-3p (Figure 3H,I). Then, to verify whether miR-137-3p could directly bind to the COL5A1 3′UTR, we carried out a dual-luciferase reporter assay. First, we designed a COL5A1-wt/mut firefly luciferase plasmid (Figure 4A) for all binding sites between miR-137-3p and the COL5A1 3′UTR, cotransfected them with the NC or miR-137-3p mimics into 293T cells, and detected the luciferase activity after 48 h. Compared with the control group, the luciferase activity in the COL5A1-wt1/2/3 and miR-137-3p mimic cotransfected groups decreased significantly, while the luciferase activity in the COL5A1-mut1/2/3 and miR-137-3p mimic cotransfected groups did not significantly change (Figure 4B). These results indicated that COL5A1 was a target gene of miR-137-3p.

### 3.6. COL5A1 Can Reversely Sponge miR-137-3p and Upregulate the Expression of FSTL1 through a ceRNA Mechanism

Studies have shown that when there are multiple MREs, the mRNAs of target genes have the potential to become miRNA sponges [32,33]. As shown in Figure 4A, we found that the 3′UTR of COL5A1 mRNA had up to three high-quality MREs that could bind to miR-137-3p. In addition, we used the expression data of 372 GC samples from TCGA to draw a dot plot (Figure S5B), from which we found that COL5A1 was more abundant in terms of molecules than the miR-137-3p. On the basis of this evidence, we speculated that COL5A1 may not only be the target gene of miR-137-3p but also the sponge of miR-137-3p, thus regulating the expression of some key genes.

Therefore, we further queried the genes that may be regulated by COL5A1 mRNA via the competing endogenous RNA (ceRNA) mechanism in the starBase database (3717 genes in total; Table S4) [34]. These genes were then intersected with the 20 hub genes mentioned above. Seven genes (FSTL1, CD44, FN1, KLF4, OCLN, PLAU, THBS2; Figure 4C) may have been regulated by COL5A1 mRNA, of which only FSTL1 and KLF4 were the target genes of miR-137-3p. Then, we used the starBase database to query the expression correlations between COL5A1 and FSTL1 (Figure 4D) or KLF4 (Figure S5C) in GC. We found that only the expression of FSTL1 and COL5A1 was significantly positively correlated (*r* = 0.805; *p* < 0.0001), while the expression of KLF4 and COL5A1 was significantly negatively correlated (*r* = −0.141; *p* = 0.0063). Thus, we speculated that COL5A1 mRNA may regulate the expression of FSTL1 through a ceRNA mechanism.

To verify this hypothesis, we detected the expression level of FSTL1 in GES-1 and four GC cell lines. As expected, we found that the expression of FSTL1 was highly consistent with COL5A1, that is, it was also high in MKN45 cells with high COL5A1 expression and low in AGS cells with low COL5A1 expression (Figure 4E,F). Moreover, the expression of FSTL1 increased after transfection of miR-137-3p inhibitor in AGS and decreased after transfection of miR-137-3p mimics in MKN45, indicating that the expression of FSTL1 was also regulated by miR-137-3p (Figure 4G,H).

Next, to verify whether miR-137-3p could directly bind to the FSTL1 3′UTR, we again carried out a dual-luciferase reporter assay. We designed an FSTL1-wt/mut firefly luciferase plasmid for the binding site of miR-137-3p and the FSTL1 3′UTR (Figure 4I) and cotransfected them with NC or miR-137-3p mimics into 293T cells. The luciferase activity was detected 48 h later. Compared with the control group, the luciferase activity in the FSTL1-wt and miR-137-3p mimic cotransfected group was significantly decreased, while that in the FSTL1-mut and miR-137-3p mimic cotransfected group was not significantly changed (Figure 4J). These results suggested that FSTL1 was a target gene of miR-137-3p.

To further confirm that the COL5A1 3′UTR could regulate the expression of FSTL1 by competitive binding to miR-137-3p through a ceRNA mechanism, we designed plasmids overexpressing the COL5A1 3′UTR (p-COL5A1 3′UTR) and plasmids overexpressing the FSTL1 3′UTR (p-FSTL1 3′UTR) and cotransfected them into 293T cells with FSTL1-wt or COL5A1-wt1/2/3 luciferase plasmid and miR-137-3p mimics, respectively. As shown in Figure 4K, compared with the control group (pCDH and FSTL1-wt cotransfected group), the luciferase activity was significantly increased in the p-COL5A1 3′UTR and FSTL1-wt cotransfected group, indicating that the COL5A1 3′UTR may sponge miR-137-3p and reduce its binding to FSTL1-wt, resulting in increased luciferase activity. However, the luciferase activity in the p-FSTL1 3′UTR and COL5A1-wt1/2/3 cotransfected group showed no significant change compared with the control group (pCDH and COL5A1-wt1/2/3 cotransfected group), indicating that the regulation of COL5A1 on FSTL1 was unidirectional, that is, FSTL1 3′UTR could not regulate the expression of COL5A1 in turn. Moreover, after overexpression of the COL5A1 3′UTR in AGS, the mRNA and protein levels of FSTL1 were significantly upregulated compared with those of the control group (Figure 4L,M), while after overexpression of the FSTL1 3′UTR, the mRNA and protein levels of COL5A1 were not significantly changed (Figure S5D,E). We also assessed the expression level of miR-137-3p in the above groups. As shown in Figure S5F, after overexpression of the COL5A1 3′UTR in AGS, the expression level of miR-137-3p was significantly downregulated, while overexpression of the FSTL1 3′UTR had no significant effect on miR-137-3p. Then, we designed three shRNA sequences for COL5A1 and confirmed that the knockdown effect of the COL5A1-sh1 sequence was optimal in MKN45 cells with high expression of COL5A1 (Figure S5G,H). We also found that after knockdown of COL5A1, the expression of miR-137-3p was significantly upregulated (Figure S5I). These results suggested that FSTL1 mRNA could not regulate COL5A1 by competitive binding to miR-137-3p. In contrast, COL5A1 mRNA could regulate FSTL1 expression by competitive binding to miR-137-3p through a ceRNA mechanism.

### 3.7. Knockdown of COL5A1 Inhibited the Proliferation, Migration, and Invasion of GC Cells, Which Can Be Rescued by miR-137-3p Inhibitor or Overexpression of FSTL1

**Figure 5:**
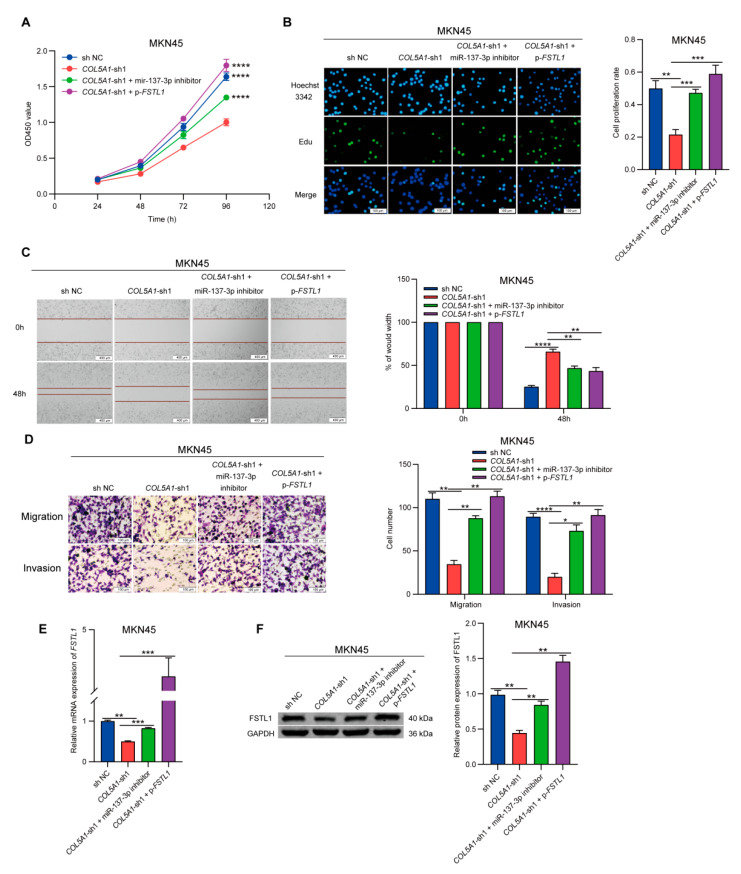
Overexpression of miR-137-3p inhibitor or FSTL1 rescued the loss of function caused by COL5A1 knockdown. CCK-8 assays (**A**) and EdU assays (**B**) were conducted to evaluate cell proliferation ability. Wound healing assays (**C**) and Transwell assays (**D**) were performed to evaluate migration and invasion ability. qRT-PCR (**E**) and Western blotting (**F**) were performed to assess the expression level of FSTL1 in the 4 groups. Full uncropped figures of Western blotting can be found in Figure S12. Data are presented as mean ± SD of three independent experiments. * *p* < 0.05; ** *p* < 0.01; *** *p* < 0.001; **** *p* < 0.0001.

To further study the effect of COL5A1 regulation of FSTL1 expression on the function of GC cells, we used COL5A1-knockdown cell lines for follow-up experiments. In addition, we designed a plasmid overexpressing FSTL1. Verification of the overexpression efficiency is shown in Figure S5J,K. The mRNA and protein levels of FSTL1 were significantly increased after transfection of AGS with the plasmid overexpressing FSTL1. Next, we designed four different experimental groups in MKN45 cells that highly expressed COL5A1 and FSTL1: the control group, the COL5A1-knockdown group, a group that underwent transfection with miR-137-3p inhibitor in the COL5A1-knockdown group, and a group that underwent transfection with plasmid overexpression of FSTL1 in the COL5A1-knockdown group. First, we assessed the cell proliferation of these four groups by CCK-8 and EdU experiments. As shown in Figure 5A,B, compared with the control group, the proliferation of MKN45 cells decreased after COL5A1 knockdown, while it was rescued after transfection of the miR-137-3p inhibitor or plasmid overexpressing FSTL1. Then, we performed wound healing and Transwell assays to detect the cell migration and invasion abilities in the four groups. Compared with the control group, the migration and invasion ability of MKN45 decreased significantly after knockdown of COL5A1, and this effect could also be rescued by transfection of miR-137-3p inhibitor or plasmid overexpressing FSTL1 (Figure 5C,D). Simultaneously, we also detected the expression level of FSTL1 in the four groups. The results showed that the expression level of FSTL1 decreased significantly after knockdown of COL5A1, while it was rescued or even higher than that in the control group after transfection of miR-137-3p inhibitor or overexpression of FSTL1 (Figure 5E,F). These findings indicated that COL5A1 could regulate the expression of FSTL1 by competitively binding to miR-137-3p to promote the proliferation, migration, and invasion of GC.

### 3.8. FSTL1 Was Related to Immune Infiltration in the TME of GC Patients

**Figure 6:**
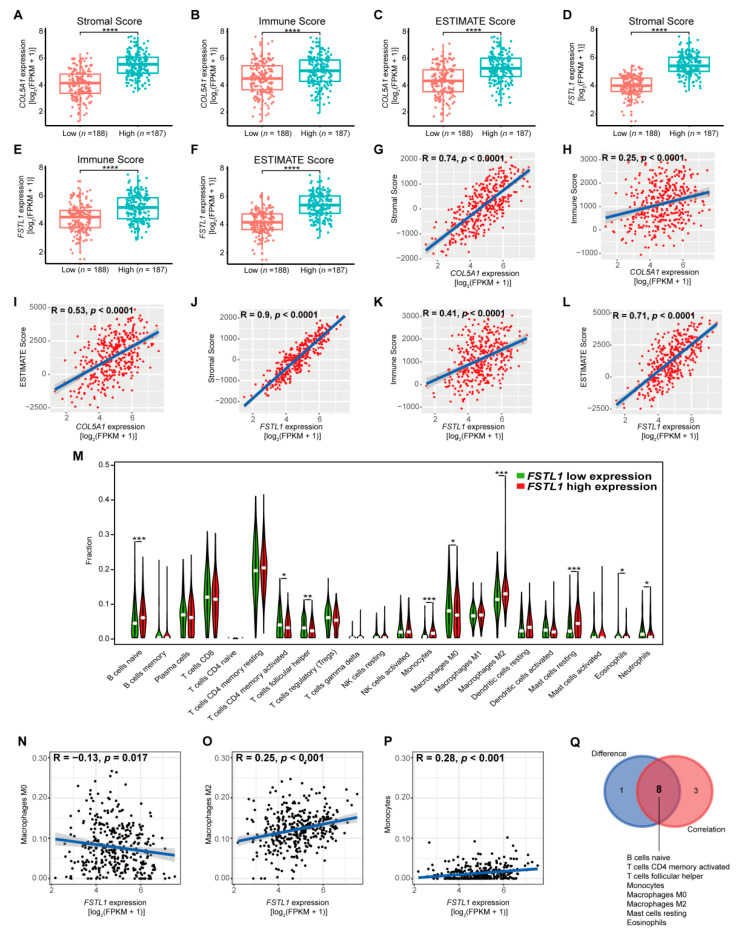
FSTL1 was related to immune infiltration in the TME of GC patients. After GC patients from TCGA were divided into high- and low-score groups (50% each) according to the stromal, immune, and ESTIMATE scores, the expression levels of COL5A1 (**A**–**C**) and FSTL1 (**D**–**F**) in the two groups were compared. Correlation analyses between COL5A1 (**G**–**I**) or FSTL1 (**J**–**L**) and the stromal, immune, and ESTIMATE scores. (**M**) GC patients from the TCGA were divided into high or low groups (50% each) according to the expression level of FSTL1, and the proportions of 22 types of immune cells in the two groups were then estimated by the CIBESORT algorithm. Correlation analyses between FSTL1 and the content of monocytes (**N**), M2 macrophages (**O**), and M0 macrophages (**P**). (**Q**) The intersection of 11 types of immune cells differed in the two groups, and 9 types of immune cells correlated with FSTL1. * *p* < 0.05; ** *p* < 0.01; *** *p* < 0.001; **** *p* < 0.0001.

We were interested in how FSTL1 regulates the progression of GC. By consulting the literature, we found that COL5A1 is related to immune infiltration in the tumor microenvironment (TME) of GC patients [35], so we wondered whether FSTL1 was also involved. Therefore, we evaluated the stromal score, immune score, and ESTIMATE score of GC patients from TCGA by the ESTIMATE algorithm and divided them into high and low groups (50% each). Then, we compared the expression levels of COL5A1 and FSTL1 in the two groups. As shown in Figure 6A–F, patients with high stromal, immune, and ESTIMATE scores also had high expression levels of COL5A1 and FSTL1. Figure 6G–L show the correlations between the expression of COL5A1 or FSTL1 and those scores. The expression of COL5A1 or FSTL1 was significantly positively correlated with the three types of scores, and the correlations between the expression of FSTL1 and those scores were significantly higher than that of COL5A1, suggesting that FSTL1 may affect tumor immune infiltration in GC patients. These results indicate that FSTL1 may promote the progression of GC by affecting tumor immunity.

## 4. Discussion

Bioinformatics analyses can effectively reveal the potential mechanism underlying the tumorigenesis and development of GC, providing new targets and ideas for the treatment of GC. In the past few years, many studies have reported the screening of key miRNAs and their target genes in GC through various bioinformatics analyses. However, with the development and updating of bioinformatics, previous methods have become outdated and not generalizable to some extent. It is worth noting that most miRNAs function by targeting coding genes, while focusing only on miRNAs and ignoring their interaction with target genes will lead to an overamplification of the functions of some miRNAs [3,4]. Combined analysis of miRNAs and their target genes can avoid this issue. In this study, we screened DEMs from the TCGA and constructed a prognostic model including miR-137-3p, which could be used to evaluate the prognosis of GC patients. Then, we predicted the target genes of 11 SRDEMs in the model and intersected them with DEGs, identifying some hub target genes through enrichment analysis, the PPI network, and WGCNA. Subsequently, we analyzed the miRNAs regulating hub genes to achieve a combined analysis of miRNAs and their target genes. Using this method, we found that miR-137-3p may play an important role in GC, and we carried out experimental verification and mechanistic exploration.

MiR-137 is located on chromosome 1p21.3, and in the non-coding gene AK094607 [36], the mature miRNA is miR-137-3p. The other mature miRNA, miR-137-5p, has not been verified [20]. Therefore, we selected miR-137-3p for further research. MiR-137-3p (i.e., miR-137) plays a tumor-suppressive role in a variety of cancers [37,38,39], including GC [40,41]. Studies have also reported that long non-coding RNAs (lncRNAs) or circular RNAs (circRNAs) sponge miR-137-3p through a ceRNA mechanism in GC [42,43]. However, no studies have reported that mRNA can regulate miR-137-3p through a ceRNA mechanism, nor has it been reported that miR-137-3p can target COL5A1 or FSTL1. In this study, we explain and supplement the phenomenon in which miR-137-3p is inhibited in GC and its tumor-suppressive role: the low expression of miR-137-3p in GC leads to high expression of its target gene, COL5A1, which in turn can sponge miR-137-3p through a ceRNA mechanism to facilitate positive feedback regulation to further reduce miR-137-3p in GC. Furthermore, from the starBase database, we learned that COL5A1 and FSTL1 shared the binding sites of 82 miRNAs, including miR-137-3p, and there were significant positive correlations between their expression levels in 31 types of tumors in the TCGA (the correlation coefficient was greater than 0.5 in 26 types of tumors) [34]. These results indicate that COL5A1 and FSTL1 may have a similar co-expression relationship in other types of tumors.

Collagen is the most abundant protein in mammals and the main component of the extracellular matrix (ECM) in the TME [44,45]. Abnormalities in collagen will affect the tumorigenesis and development of a variety of tumors as well as the infiltration of immune cells in the TME [35,46,47]. The prognostic value of multiple COL genes for various tumors has been confirmed [48,49,50,51]. Among the hub genes in this paper, COL genes accounted for a large proportion. In the WGCNA results, COL genes were mostly concentrated in the same module, suggesting that they had the same expression pattern and may play the same role in GC. In the present study, we only selected COL5A1 from COL genes for experimental verification, while other COL genes are worthy of further attention and in-depth research.

Follistatin like-1 (FSTL1) is a secretory glycoprotein that is involved in a variety of signaling pathways and biological processes, including the regulation of angiogenesis and the immune response. However, the role of FSTL1 in cancer has been controversial [52]. FSTL1 is downregulated in ovarian cancer, endometrioid cancer, and kidney carcinoma, and a decrease in FSTL1 is related to a poor prognosis [53,54]. In contrast, in esophageal squamous cell carcinoma, gastric cancer, colorectal cancer, and breast cancer, increased expression of FSTL1 is associated with a poor prognosis [55,56,57,58]. Previous studies have shown that FSTL1 is closely related to the inflammatory response [52]. Chie Kudo-Saito et al. reported that FSTL1 inhibits antitumor immunity and promotes tumor invasion as well as bone metastasis [59]. In subsequent studies, they demonstrated that FSTL1 is a determinant of immune dysfunction mediated by mesenchymal stem cells (MSCs) and immunomodulatory cells. Blocking FSTL1 could significantly inhibit tumor progression and metastasis in a mouse tumor model with increased MSCs. The expression of DIP2A (the receptor of FSTL1) in tumor cells is the key to FSTL1-induced immune resistance [60].

Our results showed that the expression of FSTL1 was closely related to immunity in the TME of GC patients. Specifically, FSTL1 was positively correlated with the stromal, immune, and ESTIMATE scores, suggesting that the expression of FSTL1 was negatively correlated with the purity of gastric cancer, while a low tumor purity often indicates a poor prognosis [61,62]. In addition, we divided GC patients from the TCGA into high/low groups (50% each) according to the expression of FSTL1. The proportions of 22 types of immune cells in the TME of each GC patient was estimated by the CIBERSORT algorithm. As shown in Figure 6M, there were differences in the contents of nine types of immune cells between the two groups. We then combined the expression of FSTL1 with the content of various immune cells for correlation analysis, from which we found that 11 types of immune cells were correlated with the expression of FSTL1 (Figure 6N–P and Figure S7). Finally, we intersected these 11 types of immune cells and the nine types of immune cells mentioned above, identifying eight types of immune cells related to FSTL1 expression (Figure 6Q), namely, naive B cells, activated memory CD4+ T cells, follicular helper T cells, monocytes, M0 macrophages, M2 macrophages, resting mast cells, and eosinophils.

Tumor-associated macrophages (TAMs) are an important type of immunosuppressive cell. Generally, monocytes and macrophages (M0 macrophages) can be polarized into classically activated macrophages (M1 macrophages) or alternatively activated macrophages (M2 macrophages) under different stimuli, and their functions are almost antagonistic to each other [63,64,65]. M1 macrophages promote the immune response and normalize irregular tumor vascular networks to sensitize cancer cells to chemotherapy and radiotherapy [66]. In contrast, TAMs are usually similar to M2 macrophages, which play an immunosuppressive role in promoting tumor progression, metastasis, and chemoresistance [67,68,69,70]. Our results showed that the expression of FSTL1 was negatively correlated with the proportion of M0 macrophages (Figure 6N) and positively correlated with the proportion of M2 macrophages (Figure 6O) and monocytes (Figure 6P). Therefore, we speculated that FSTL1 could recruit monocytes and macrophages or promote the polarization of monocytes and M0 macrophages into M2 macrophages. This phenomenon may be related to the fact that FSTL1 is a secretory glycoprotein and the interaction between FSTL1 and its receptor DIP2A [60]. This hypothesis and the specific underlying mechanism require further study and verification.

## 5. Conclusions

MiR-137-3p played a tumor-suppressive role in GC, and its target gene COL5A1 could reversely sponge miR-137-3p to relieve its targeted inhibition of FSTL1, which might promote the progression of GC by affecting immune infiltration.

## Figures and Tables

**Table 1:** Univariate Cox regression analysis of the 18 miRNAs associated with survival in GC patients.

miRNA	HR	*p*-Value
hsa-miR-7-2	0.84 (0.75–0.93)	<0.001
hsa-miR-328	1.26 (1.09–1.47)	0.002
hsa-miR-3923	1.24 (1.08–1.43)	0.002
hsa-miR-675	1.10 (1.03–1.16)	0.003
hsa-miR-7-3	0.86 (0.78–0.96)	0.006
hsa-miR-549a	0.84 (0.74–0.96)	0.009
hsa-miR-125a	1.27 (1.06–1.52)	0.009
hsa-miR-708	1.16 (1.03–1.31)	0.017
hsa-miR-217	1.12 (1.02–1.24)	0.018
hsa-miR-137	1.14 (1.02–1.27)	0.018
hsa-miR-100	1.12 (1.02–1.23)	0.019
hsa-miR-2115	0.82 (0.69–0.98)	0.029
hsa-miR-548v	1.14 (1.01–1.28)	0.029
hsa-miR-6511b-1	1.20 (1.01–1.42)	0.034
hsa-miR-187	1.07 (1.00–1.13)	0.035
hsa-miR-145	1.10 (1.00–1.20)	0.043
hsa-miR-371a	1.11 (1.00–1.24)	0.045
hsa-miR-216a	1.11 (1.00–1.24)	0.049

**Table 2:** Two hundred thirty-three DETGs shared by DEGs and the target genes of 11 SRDEMs.

SRDEM	DETGs
hsa-miR-328	*ESRP1*, ***IFIT1***, *MICALL1*, *MLLT11*, *MN1*, *PRDM16*
hsa-miR-549a	***ATP11A***, *C1R*, ***CDH11***, *EPPK1*, ***FUT9***, *GLUL*, ***GPD1L***, *LAPTM5*, ***NIPAL1***, ***OCLN***, *PPP2R3A*, ***PRELID2***, *RAI14*, ***SGK1***, ***SH3BGRL2***, *SOX21*, *ST3GAL6*, ***SYNJ2BP***, ***TFCP2L1***, *THBS2*
hsa-miR-708	*AADAC*, ***ABHD2***, ***AHCYL2***, ***ANK3***, *AQP4*, *BCL6*, *CAP2*, ***CCL28***, ***CCND2***, ***CDH2***, ***CM**TM4*, *COL10A1*, *EMP1*, *EPB41L3*, *ETNK1*, ***FGD4***, *FUT2*, *GABRP*, ***GPR155***, ***IFIT1***, ***KIAA1958***, *MAGI3*, ***MAP1B***, *NLRP1*, *PAFAH2*, *PLSCR1*, *PTGER3*, *REPS2*, *RGN*, ***RHOBTB1***, *SCRG1*, ***SIK2***, ***STS***, ***TMEM161B***, ***UGT8***, *XK*, ***ZEB1***, *ZNF462*
hsa-miR-217	***ABCC9***,***ANK3***, *ANLN*, ***ATP1B1***, *CALD1*, ***CORO1C***, ***ESRRG***, *FN1*, ***HOMER2***, ***SEMA3A***, ***SLC4A4***, *TMEM246*, *UBL3*
hsa-miR-371a	***ABHD2***,***CALU***, ***ELOVL6***, *HAND2*, ***MSRB3***, ***NEUROD1***, ***OCLN***, *SGPP2*, ***SH3BGRL2***, ***SLC16A7***, ***SYNJ2BP***, *TMEM185B*, *TOB1*
hsa-miR-7-2	*BUB1*, ***CALU***, *COL2A1*, *CTSB*, ***DSP***, ***EHF***, ***EPB41L3***, ***ESRRG***, ***GALNT3***, *GATA6*, *GATM*, *GJA1*, ***GJC1***, *GREM1*, ***HOMER2***, *IL20RA*, ***KLF4***, ***MAP1B***, *NTN4*, ***PDE4B***, *PDZRN4*, ***PRKCB***, ***PTGER3***, ***SGK1***, ***SH3BGRL2***, *SKAP2*, ***SLC16A7***, ***SLC4A4***, *SPP1*, ***SYNJ2BP***, ***TCF4***
hsa-miR-675	***ABCC9***, *ANKRD22*, ***ATP11A***, *C6*, ***CDH11***, *CDH13*, *COBLL1*, *COL5A2*, *COLCA1*, ***CORO1C***, *DMRTA1*, *GC*, *ISPD*, *MYBL1*, *PIGR*, ***PIP5K1B***, *SERPINF1*, ***SLC12A2***, ***SMOC2***, ***STS***, *TFEC*, ***UGT8***, *USP53*
hsa-miR-137	***ABCC9***,***ABHD2***, ***AHCYL2***, *AJUBA*, *ANGPTL2*, ***ATP1B1***, ***CNTN3***, *COL5A1*, ***CXADR***, ***DSP***, ***DUSP10***, *DUSP4*, *EHBP1L1*, ***ELOVL6***, ***ESRRG***, ***FAT3***, *FBXO32*, ***FGD4***, *FGL2*, *FRMD6*, ***FSTL1***, ***FUT9***, *GFPT1*, ***GJC1***, *GPX7*, *GULP1*, ***KIAA1958***, *KLF15*, ***KLF4***, *LBH*, *LCP2*, *MPC1*, ***MSRB3***, ***NEUROD1***, ***NIPAL1***, *NT5DC2*, *PDLIM3*, *PKDCC*, *PLEKHO2*, *RCN3*, *RFTN1*, ***RHOBTB1***, *RRAGD*, *SERPINA3*, ***SIK2***, ***SIPA1L2***, ***SLC12A2***, *TBC1D1*, ***TCF4***, *THBS4*, *TMEM125*, *TMEM56*, ***TRPS1***, ***TWIST1***, *YBX3*, *ZNF385B*
hsa-miR-548v	***ABHD2***, *APOBEC1*, ***ATP1B1***, ***CCND2***, ***CDH2***, *CHN1*, *CLDN1*, ***CMTM4***, ***FUT9***, ***GALNT3***, *GATA4*, ***GPSM2***, *HOXB7*, *MAP4K4*, *MRAP2*, *NAT1*, *NNT*, *PRKACB*, *PRSS8*, *PTPRZ1*, ***RAB27B***, ***SLC16A7***, ***SLC4A4***, *SYNC*, *TMEM92*, *TNFSF13B*
hsa-miR-2115	*ABCC5*, *ADAM28*, *ALCAM*, *ALDH6A1*, *ARSD*, ***ATP11A***, *BCAS1*, *BEX5*, *C6orf58*, *CA8*, ***CCL28***, *CD44*, *CDS1*, *CENPF*, *CEP170*, *CKS1B*, ***CNTN3***, *COL11A1*, ***CORO1C***, *CRISPLD1*, *CSGALNACT2*, ***CXADR***, *CXCL9*, *DAB2*, ***DSP***, ***DUSP10***, ***EHF***, *EMILIN2*, ***EMP1***, *FAM83F*, ***FAT3***, *FGF7*, *FOXA1*, ***FSTL1***, *FUT8*, ***FUT9***, *GALNT6*, *GNB4*, ***GPD1L***, ***GPR155***, ***GPSM2***, *GSTA1*, *GSTA2*, *HOOK1*, *HOXB6*, *IGF1*, ***KL***, *LRRC17*, *NEURL1B*, *NID2*, *NQO1*, *NSUN7*, ***PDE4B***, *PEA15*, *PLAU*, *PMP22*, ***PRELID2***, ***PRKCB***, *PTPN3*, *PTPRN2*, *RAB11FIP2*, ***RAB27B***, *RAP1GAP*, ***RHOBTB1***, ***SEMA3A***, ***SGK1***, *SH3GL2*, ***SIK2***, ***SIPA1L2***, ***SLC16A7***, ***SMOC2***, *SPARC*, *SULF1*, ***SYNJ2BP***, ***TFCP2L1***, ***TMEM161B***, *TMEM229A*, ***TRPS1***, *TSPAN12*, ***TWIST1***, ***UGT8***, ***ZEB1***
hsa-miR-3923	***ATP1B1***, *DSC2*, *GKN1*, *GRIA4*, ***KL***, *LIFR*, *MSR1*, ***NEUROD1***, *ODAM*, ***PIP5K1B***, *PTGS2*, *SAMD13*, *SOSTDC1*

Genes in bold font are targeted by more than 1 SRDEM.

## Data Availability

Data are contained within the article and supplementary files.

## Supplementary Materials

The following supporting information can be downloaded at https://www.mdpi.com/article/10.3390/cancers14133244/s1, Table S1: A list of downloaded samples from TCGA. Table S2: qRT-PCR primer sequences used in this study. Table S3: The top 5 modules containing the most DEGs and DETGs. Table S4: Genes that might be regulated by COL5A1 via ceRNA mechanism in the starBase database. Figure S1: Target genes of 11 SRDEMs. Figure S2: The regulation network consists of 11 SRDEMs and 233 DETGs. Figure S3: Functional enrichment analysis of 233 DETGs. Figure S4: Weighted gene co-expression analysis. Figure S5: Efficiency of knockdown or overexpression. Figure S6: Correlation analyses between FSTL1 and the content of immune cells. Figure S7: Full uncropped figures of Western blotting for Figure 3C. Figure S8: Full uncropped figures of Western blotting for Figure 3I. Figure S9: Full uncropped figures of Western blotting for Figure 4F. Figure S10: Full uncropped figures of Western blotting for Figure 4H. Figure S11: Full uncropped figures of Western blotting for Figure 4M. Figure S12: Full uncropped figures of Western blotting for Figure 5F. Supplementary Informations: The code of differential expression analysis.
