# Supplementary material for: COL5A1 Promotes the Progression of Gastric Cancer by Acting as a ceRNA of miR-137-3p to Upregulate FSTL1 Expression
Source: Cancers (Basel). 2022 Jul 1;14(13):3244. doi: 10.3390/cancers14133244 (PMC9264898; doi:10.3390/cancers14133244)
Supplement: Supplementary file 1 [file cancers-14-03244-s001.zip › cancers-1683740 - supplementary figures.pdf]

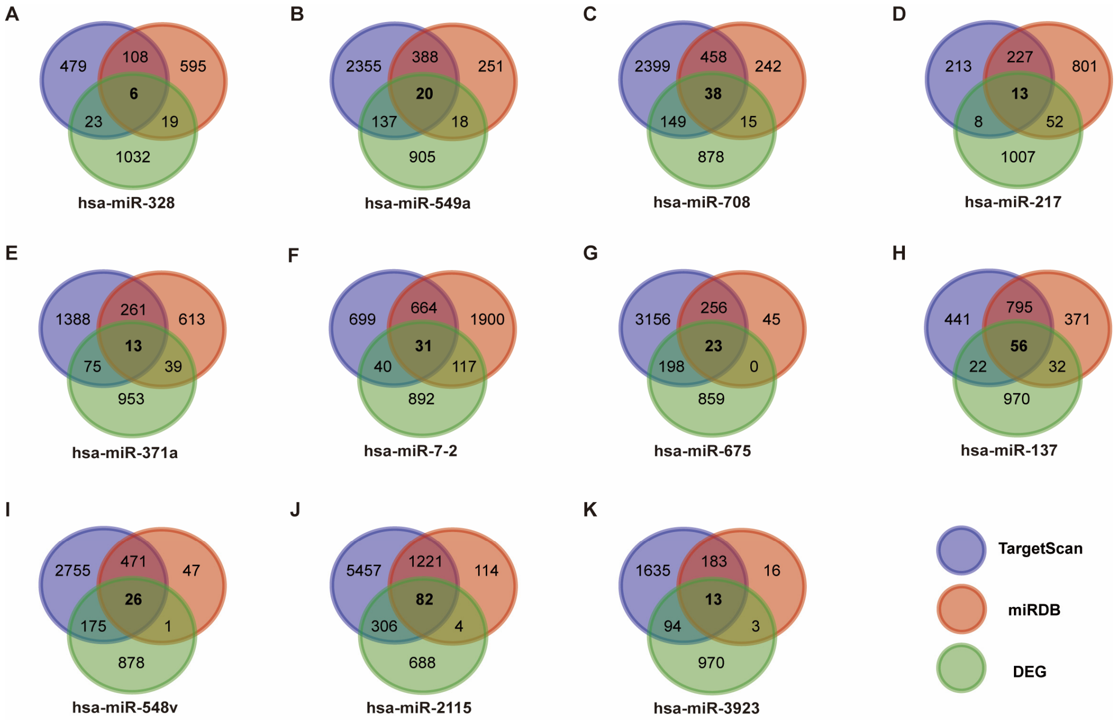

Figure S1. Target genes of 11 SRDEMs. Blue represents genes predicted by TargetScan, red represents genes predicted by miRDB and green represents DEGs.

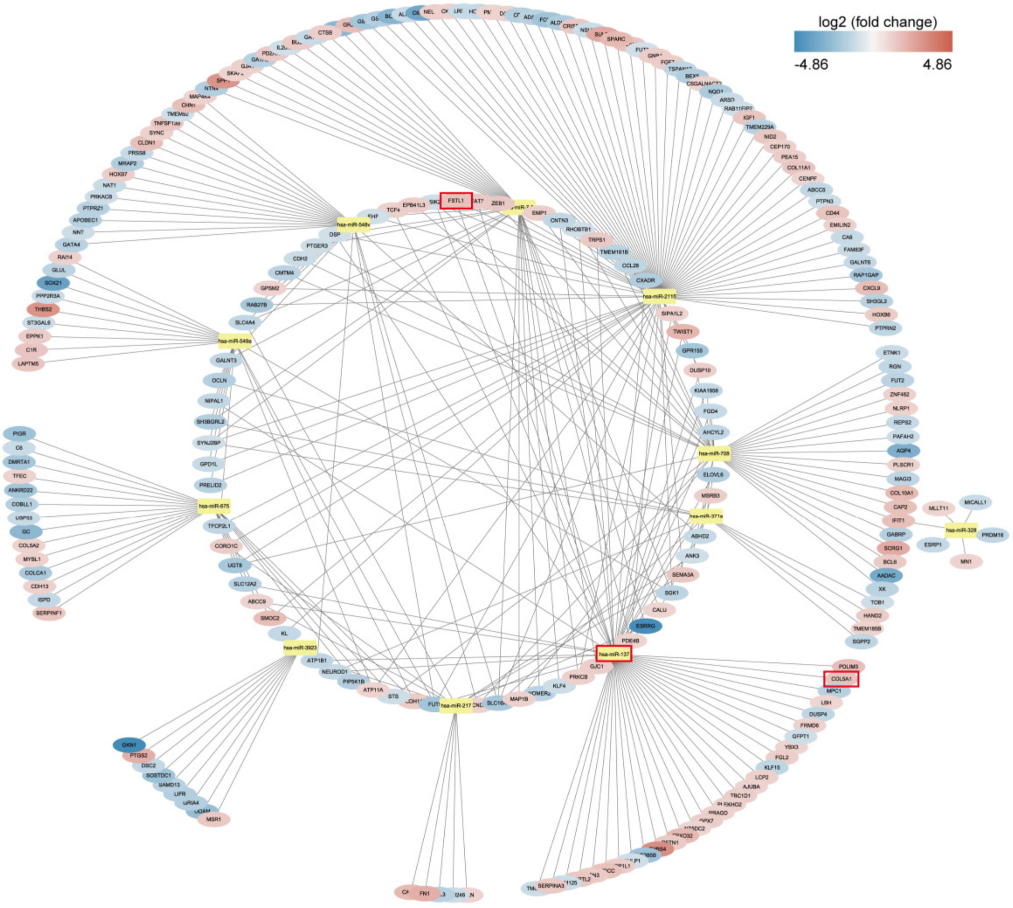

Figure S2. The regulation network consists of 11 SRDEMs and 233 DETGs**.** Yellow represents SRDEMs, red and blue represents DETGs. The color of the node deepens as the value of $|\log_2 FC|$ increases. In this figure, COL5A1, FSTL1 and miR-137-3p are highlighted.

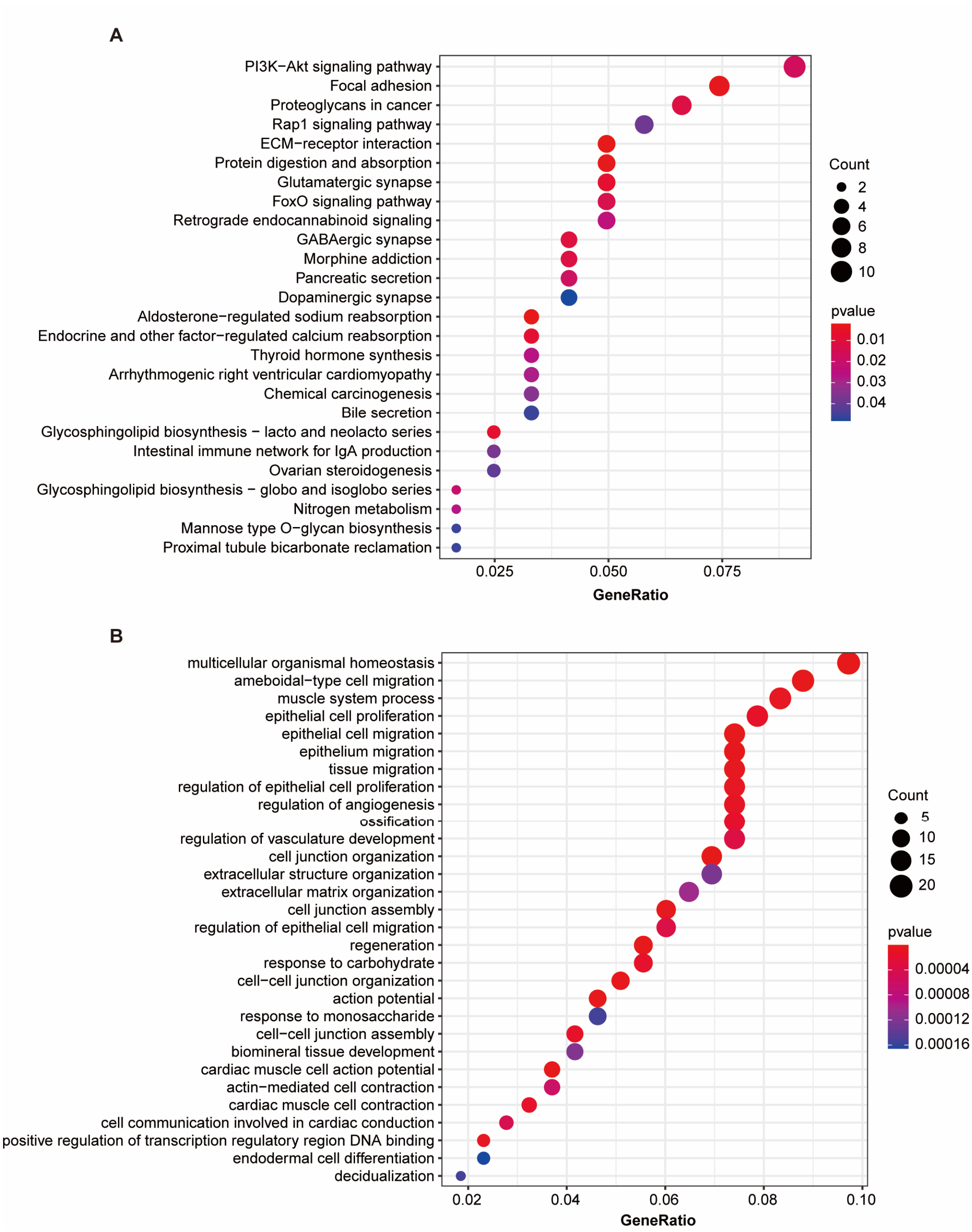

Figure S3. Functional enrichment analysis of 233 DETGs. (**A**) KEGG pathway enrichment analysis. (**B**) GO enrichment analysis. The x-axis represents the gene ratio (the proportion of genes enriched in a pathway to the total number of genes in this pathway), and the y-axis represents different KEGG pathways and GO terms. The size of the bubbles grows as the number of involved genes increases, and the p value is represented by the color of the bubbles.

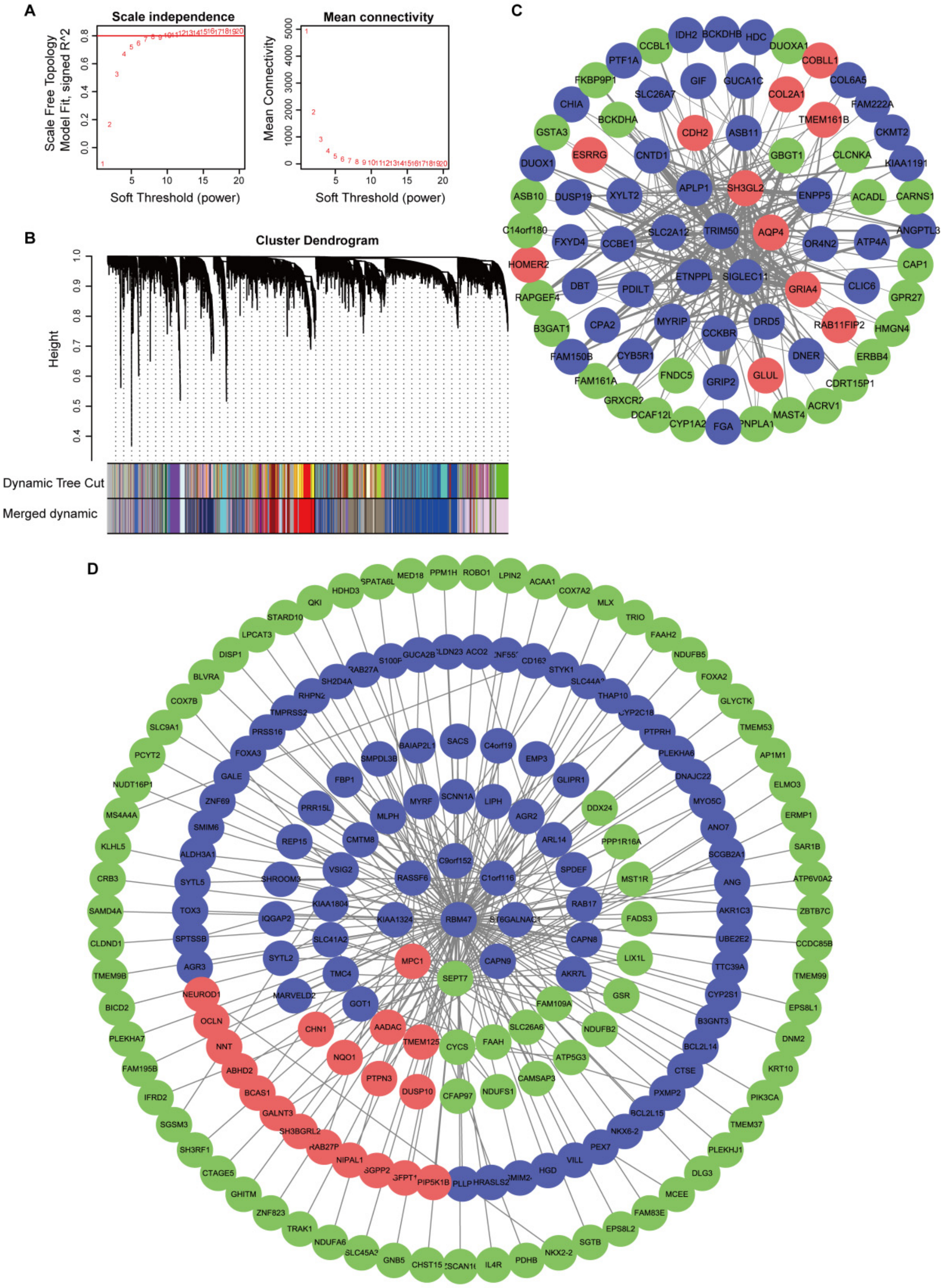

Figure S4. Weighted gene co-expression analysis. (**A**) Determination of the optimal soft threshold in WGCNA. (**B**) Cluster dendrogram of all the genes in GSE118916. Each leaf represents a separate gene, and each branch represents a co-expression gene module. (**C**) Blue module. (**D**) Bisque4 module. Blue represents DEGs, red represents DETGs and green represents other genes.

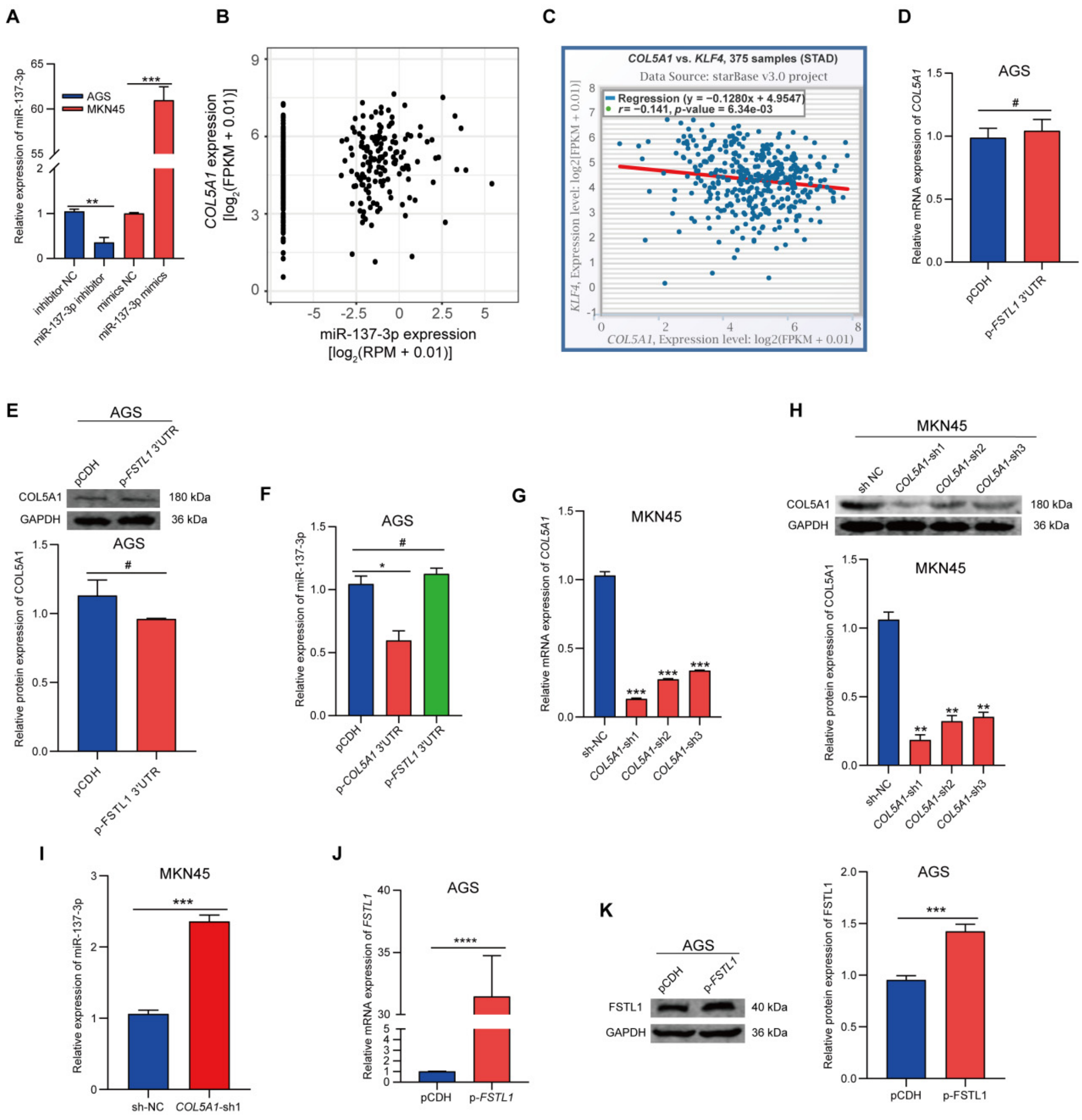

Figure S5. Efficiency of knockdown or overexpression. (**A**) The expression level of miR-137-3p was detected by qRT-PCR after transfection of miR-137-3p mimics or inhibitor. (**B**) Dot plot between the expression of *COL5A1* and miR-137-3p in GC from TCGA (372 samples included). (**C**) Correlation analysis between *COL5A1* and *KLF4* in GC from TCGA. The expression level of *COL5A1* was detected by qRT-PCR (D) and western blotting (E) after transfection with the *FSTL1* 3′UTR in AGS cells. The expression level of miR-137-3p was detected by qRT-PCR after transfection with the *COL5A1* 3′UTR or *FSTL1* 3′UTR in AGS cells (F). qRT-PCR (G) and western blotting (H) were used to detect the mRNA and protein levels of *COL5A1* after knockdown of *COL5A1*. The expression level of miR-137-3p was detected by qRT-PCR after knockdown of *COL5A1* in MKN45 cells (I). The expression level of *FSTL1* was detected by qRT-PCR (J) and western blotting (K) after transfection of plasmid overexpressing *FSTL1*. Data are presented as mean ± SD of three independent experiments. # $p > 0.05$; * $p < 0.05$; ** $p < 0.01$; *** $p < 0.001$; **** $p < 0.0001$.

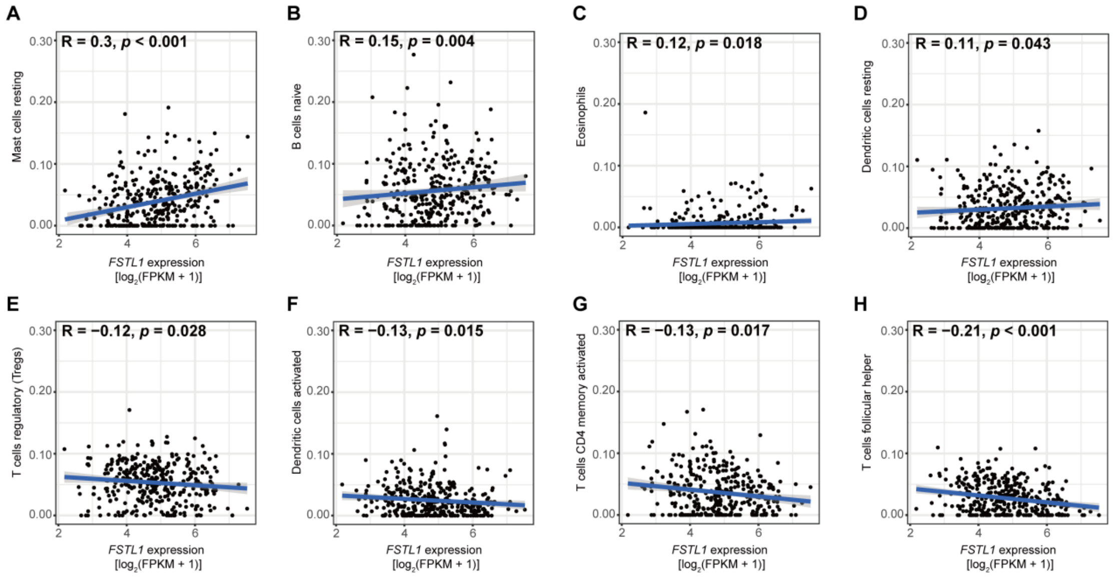

Figure S6. Correlation analyses between FSTL1 and the content of immune cells**.** (**A**) Resting mast cells. (B) Naive B cells. (**C**) Eosinophils. (**D**) Resting dendritic cells. (**E**) Regulatory T cells. (**F**) Activated dendritic cells. (**G**) Activated memory CD4+ T cells. (**H**) Follicular helper T cells.

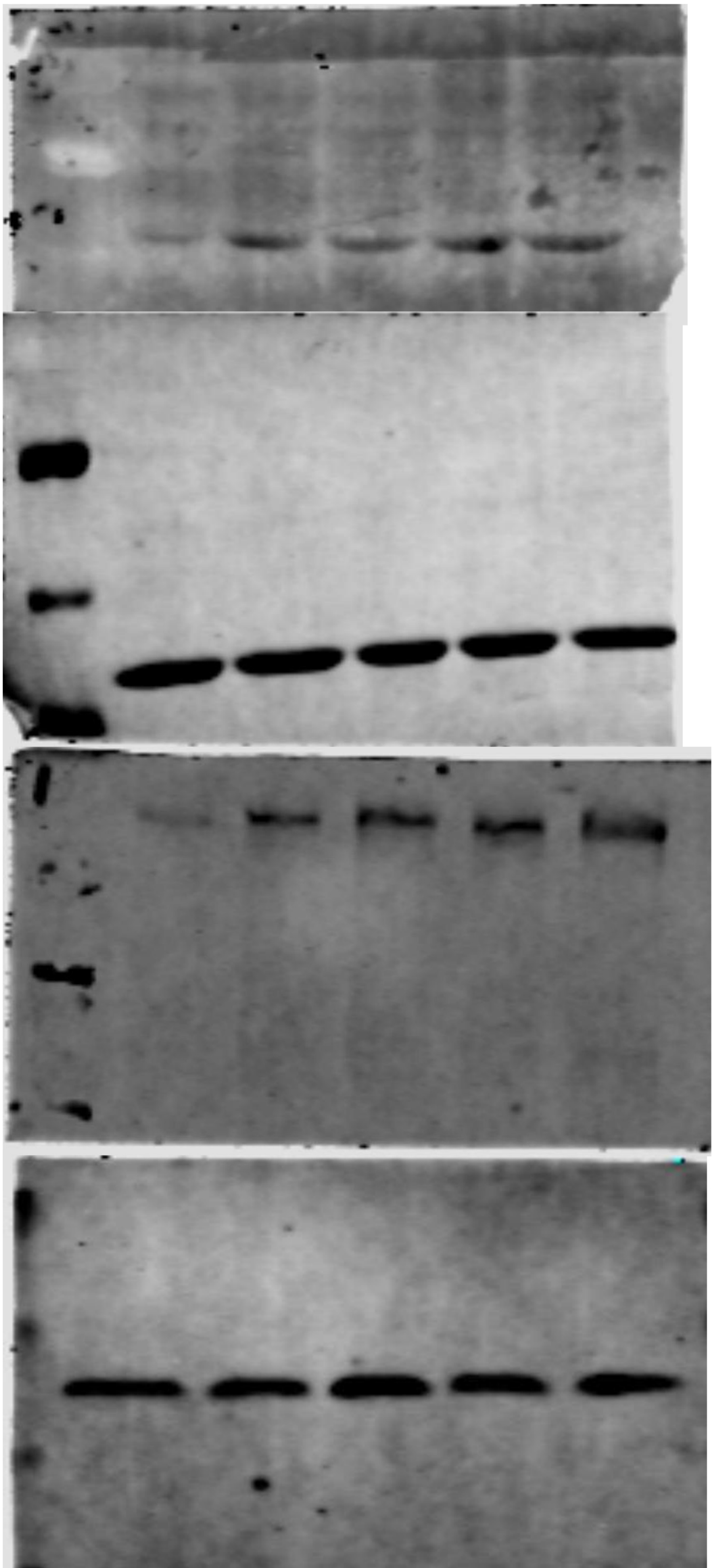

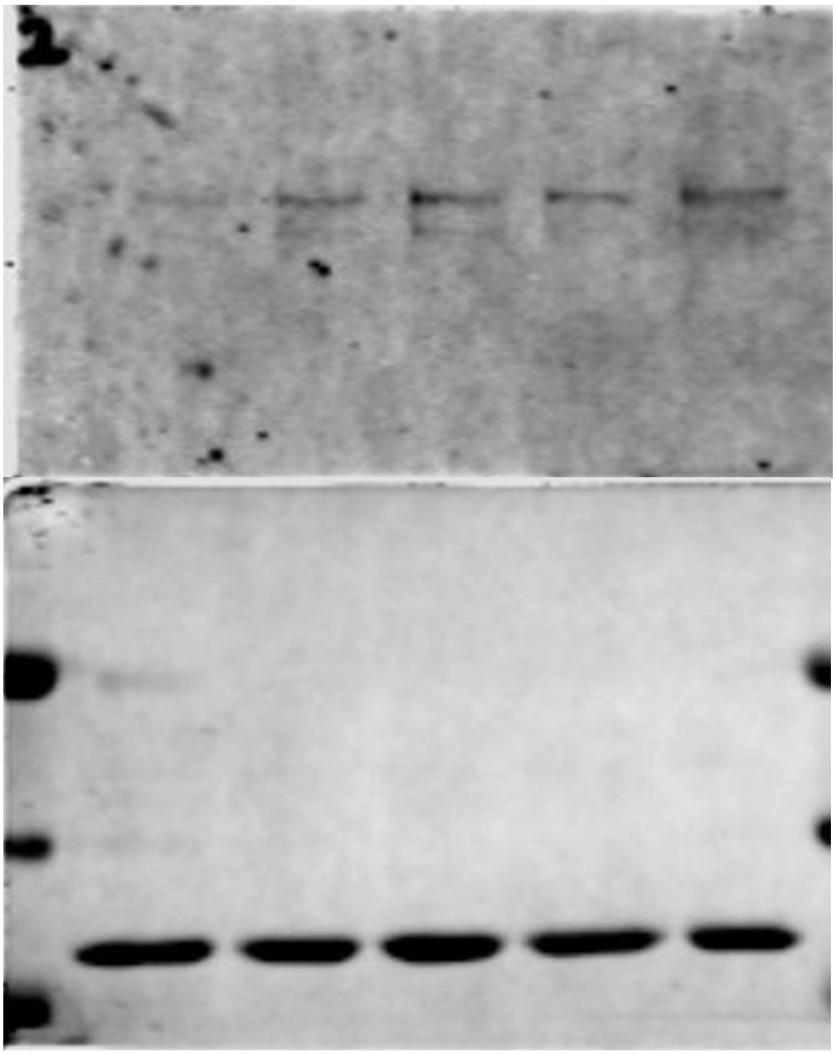

Figure S7. Full uncropped figures of Western blotting for Figure 3C.

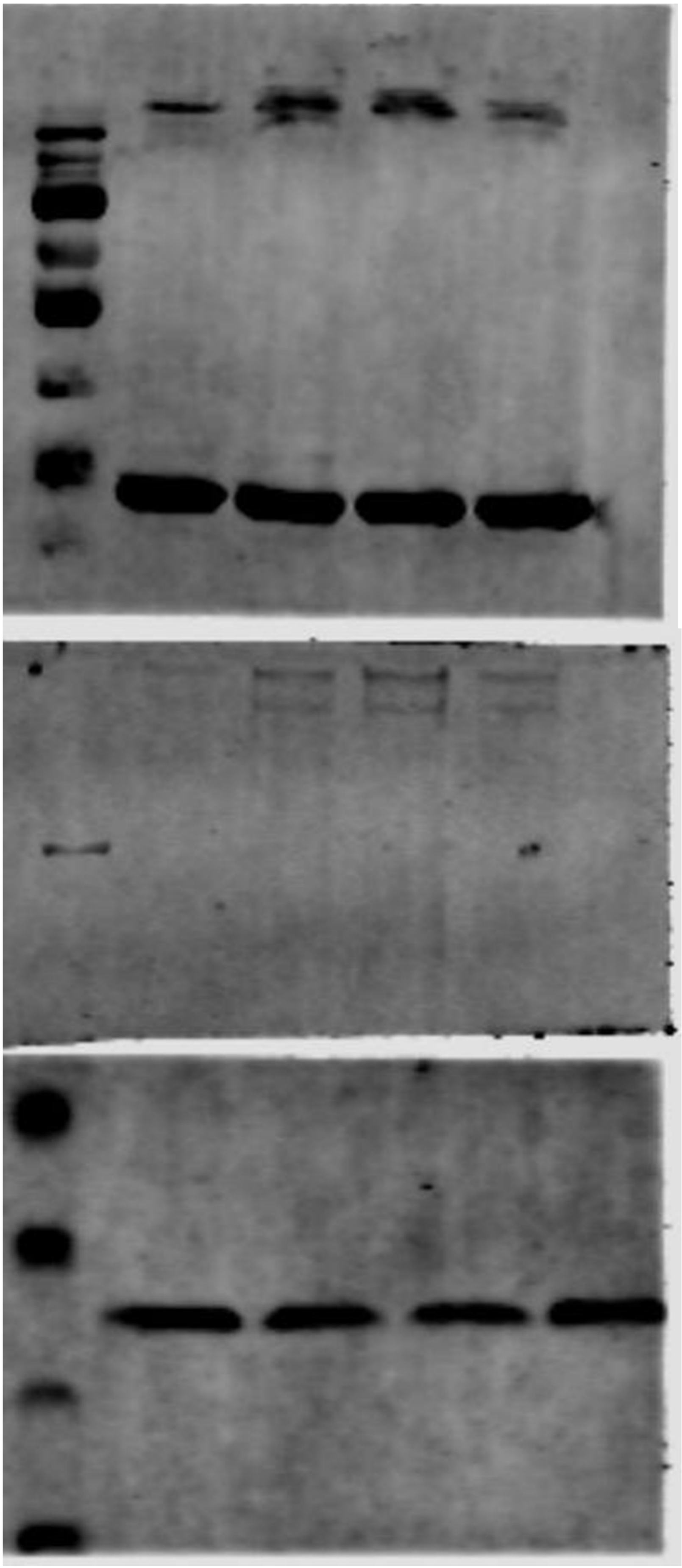

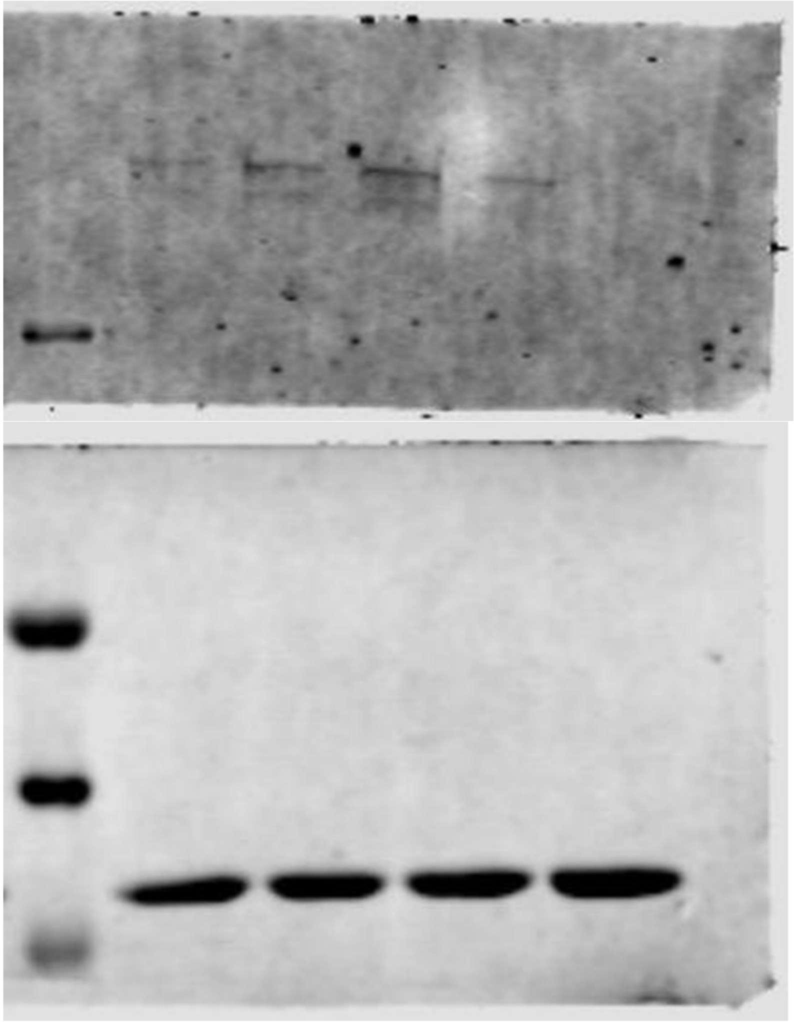

Figure S8. Full uncropped figures of Western blotting for Figure 3I.

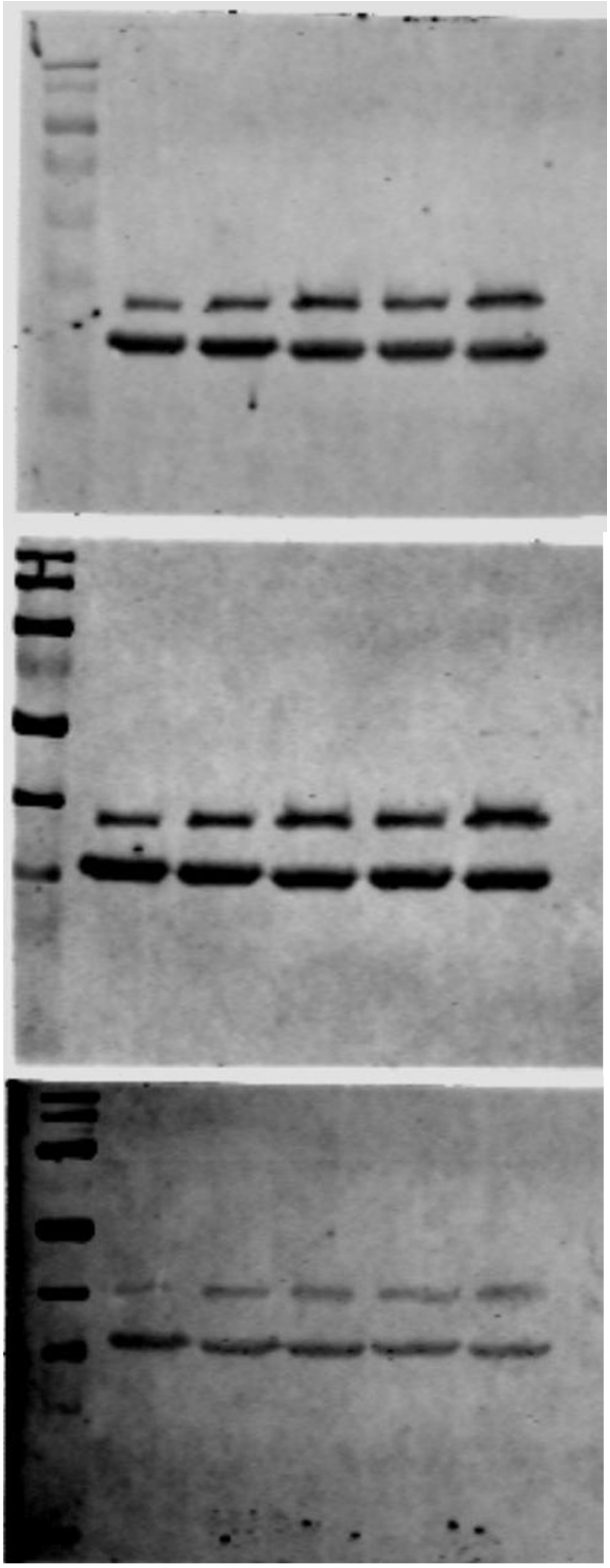

Figure S9. Full uncropped figures of Western blotting for Figure 4F.

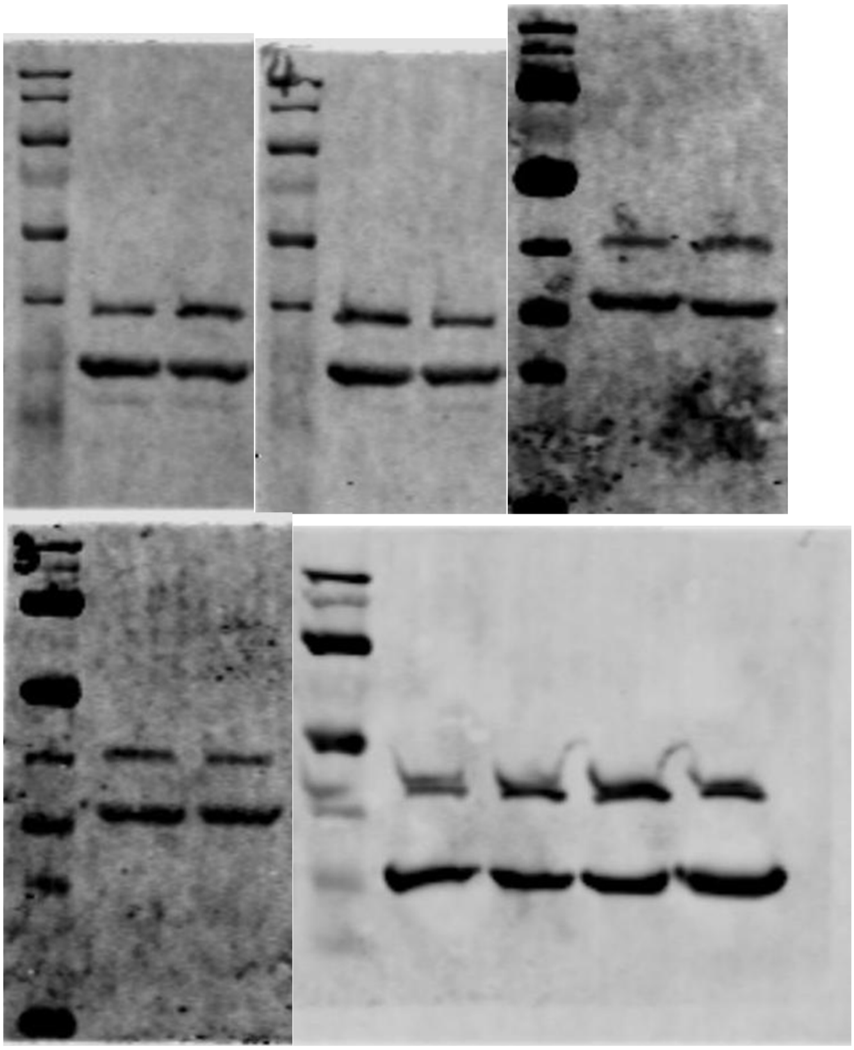

Figure S10. Full uncropped figures of Western blotting for Figure 4H.

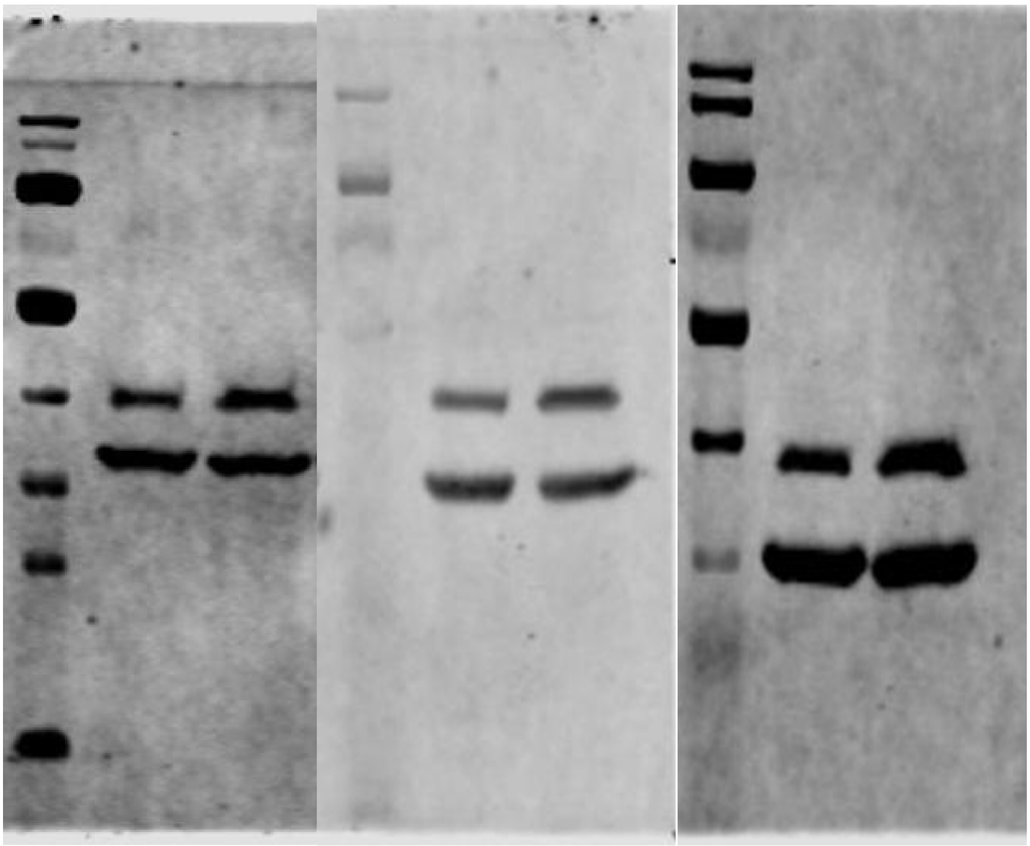

Figure S11. Full uncropped figures of Western blotting for Figure 4M.

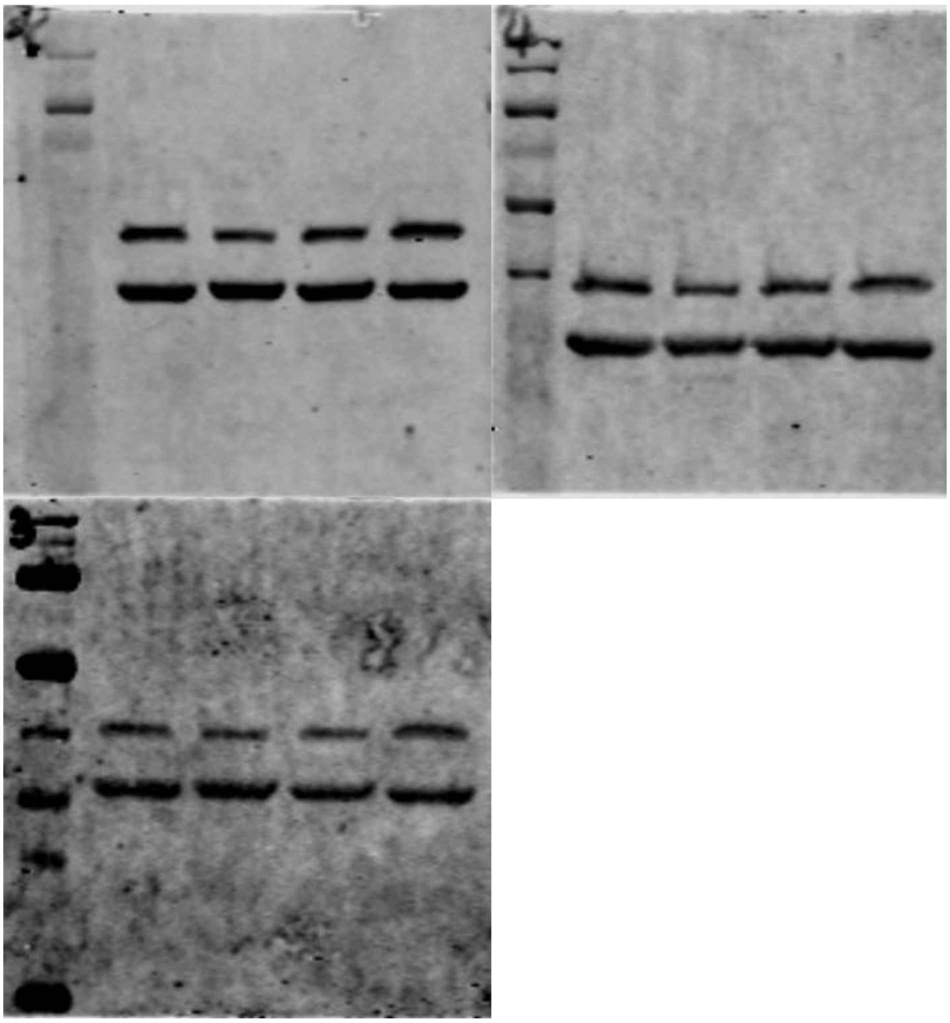

Figure S12. Full uncropped figures of Western blotting for Figure 5F.

Supplementary Information:

```
##########code for differential analysis of miRNAs from TCGA########

#Load library
library(edgeR)

#Read data
tcga<-read.table("miRNAmatrix.txt",header = T,sep = "\t",check.names = F)
tcga=as.matrix(tcga)
rownames(tcga)=tcga[,1]
GeneExp=tcga[,2:ncol(tcga)]
TCGA=matrix(as.numeric(as.ma-
trix(GeneExp)),nrow=nrow(GeneExp),dimnames=list(rownames(GeneExp),colnames(Ge
neExp)))

#Same samples were averaged
TCGA=avereps(TCGA)

#Filter out samples with low expression
TCGA=TCGA[rowMeans(TCGA)>1,]

#Calculate the number of each group and set the comparison groups
mitumor <- colnames(TCGA)[as.integer(substr(colnames(TCGA),14,15)) < 10]
minormal <- colnames(TCGA)[as.integer(substr(colnames(TCGA),14,15)) >= 10]
design=c(rep("normal",length(minormal)),rep("tumor",length(mitumor)))
mydesign <- model.matrix(~design)
mydgelist <- DGEList(counts=TCGA,group=design)

#Normalization and differential analysis
mydgelist<- calcNormFactors(mydgelist)
mydgelist<- estimateCommonDisp(mydgelist)
mydgelist <- estimateTagwiseDisp(mydgelist,trend = "movingave")

#Two tailed test was performed
mytest <- exactTest(mydgelist,pair = c("normal","tumor"))
Allgene<-topTags(mytest,n=10000000)
Allgene=Allgene$table
iddata<-mydgelist$pseudo.counts

#Output results
write.table(Allgene,"Allgene.xls",sep="\t",quote = F)
Diffgene = Allgene[(Allgene$FDR < 0.05 & (Allgene$logFC>1 | Allgene$logFC<(-
1))),]
write.table(Diffgene, "Diffgene.xls",sep="\t",quote=F)



###########code for differential analysis of GSE118916############

#Load library
library(limma)

#Read data
geo <- read.table("GSE118916.txt",sep = "\t",row.names = 1, header = T)
```

```
#Cutoff value
logFCfilter=1
adjPfilter=0.05

#Set the comparison groups
conNum=15
treatNum=15
Type=c(rep("tumor",treatNum),rep("normal",conNum))
design <- model.matrix(~0+factor(Type))
colnames(design) <- c("normal","tumor")

#Differential analysis
fit <- lmFit(geo,design)
cont.matrix<-makeContrasts(tumor-normal,levels=design)
fit2 <- contrasts.fit(fit, cont.matrix)
fit2 <- eBayes(fit2)

#Output results
allDiff=topTable(fit2,adjust='fdr',number=200000)
write.table(allDiff,file="all.xls",sep="\t",quote=F)
diffSig=allDiff[with(allDiff, (abs(logFC)>logFCfilter & adj.P.Val < adjPfilter )), ]
diffSigOut=rbind(id=colnames(diffSig),diffSig)
write.table(diffSigOut,file="diff.xls",sep="\t",quote=F,col.names=F)
```
